# A Nucleus-Imaging Probe That Selectively Stabilizes a Minor Conformation of *c-MYC* G-quadruplex and Down-regulates *c-MYC* Transcription in Human Cancer Cells

**DOI:** 10.1038/srep13183

**Published:** 2015-08-19

**Authors:** Deepanjan Panda, Manish Debnath, Samir Mandal, Irene Bessi, Harald Schwalbe, Jyotirmayee Dash

**Affiliations:** 1 Department of Organic Chemistry, Indian Association for the Cultivation of Science, Jadavpur, Kolkata-700032, India; 2 Institute of Organic Chemistry and Chemical Biology, Goethe University Frankfurt and Centre for Biomolecular, Magnetic Resonance, Max-von-Laue Strasse 7, 60438, Frankfurt am Main, Germany

## Abstract

The *c-MYC* proto-oncogene is a regulator of fundamental cellular processes such as cell cycle progression and apoptosis. The development of novel *c-MYC* inhibitors that can act by targeting the *c-MYC* DNA G-quadruplex at the level of transcription would provide potential insight into structure-based design of small molecules and lead to a promising arena for cancer therapy. Herein we report our finding that two simple bis-triazolylcarbazole derivatives can inhibit *c-MYC* transcription, possibly by stabilizing the *c-MYC* G-quadruplex. These compounds are prepared using a facile and modular approach based on Cu(I) catalysed azide and alkyne cycloaddition. A carbazole ligand with carboxamide side chains is found to be microenvironment-sensitive and highly selective for “turn-on” detection of *c-MYC* quadruplex over duplex DNA. This fluorescent probe is applicable to visualize the cellular nucleus in living cells. Interestingly, the ligand binds to *c-MYC* in an asymmetric fashion and selects the minor-populated conformer via conformational selection.

The *c-MYC* oncogene is overexpressed in a broad spectrum of human malignancies and emerged as a potential therapeutic target for cancer treatment^1^. The overexpression of *c-MYC* in hepatic cells is frequently associated with the development of hepatocellular carcinoma^2^. Small molecule mediated inhibition of *c-MYC* imparts growth arrest in liver cancer cells and even down-regulates the human telomerase reverse transcriptase (hTERT) activity^3^,^4^. Therefore *c-MYC* is an attractive target in developing new therapies for hepatocellular carcinoma. Transcription of *c-MYC* is primarily regulated by a 27 base guanine-rich sequence present within the nuclease hypersensitivity element III1 (NHE III1)^5^. This sequence, located -142 to -115 bp upstream of the P1 promoter of *c-MYC* oncogene, exists in equilibrium between transcriptionally active forms (double helical and single stranded) and a silenced form, which is able to fold into a G-quadruplex structure^6^. The 27-mer sequence responsible of *c-MYC* regulation contains five guanine runs and it has been shown that in K^+^-containing solution only the four consecutive 3′ G-runs are involved in the formation of the major G-quadruplex structure which causes the gene transcriptional silencing^7^,^8^. However, the major G-quadruplex formed under superhelicity conditions involves the four consecutive 5′ G-runs^9^,^10^. Various G-quadruplex structures derived from different G-rich tracts of the *c-MYC* regulatory element NHE III1 have been reported^11^,^12^,^13^. The pioneering work of Siddiqui-Jain *et al.* showed that small molecules stabilizing the *c-MYC* G-quadruplex can reduce *c-MYC* transcription in cancer cells^14^. Since then, several classes of small molecules, which can bind and stabilize *c-MYC* quadruplex have been developed^15^,^16^,^17^,^18^,^19^,^20^,^21^,^22^,^23^,^24^,^25^,^26^,^27^ and few of them have been structurally characterized in complex with *c-MYC G-*quadruplex by NMR^12^,^24^. However, only a few molecules, for example, cationic porphyrins^20^, quindolines^21^ and metal complexes^25^ have been taken forward to cellular system to exert their desired biological effect^15^. In most cases, either the ligand has been prepared using multistep syntheses with low overall yields making it not readily accessible or the mode of binding to the *c-MYC* quadruplex remain unknown. Therefore, it is important to devise easily synthesizable and cost-effective *c-MYC* stabilizers which can exhibit tailored antiproliferative activities in cancer cells.

Carbazole derivatives exhibit a wide range of pharamacological activities. A few carbazole derivatives have been reported to bind to *h-TELO* G-quadruplex^27^,^28^. Herein, we delineate a modular synthetic access to novel bis-triazolyl carbazole derivatives^29^ as potent “turn on” G-quadruplex probes, which bind to *c-MYC* quadruplex *via* conformational selection with the potential to down-regulate *c-MYC* transcription in hepatocellular carcinoma cells.

## Results

### Modular synthesis of bis-triazolyl carbazole ligands

**Figure 1:**
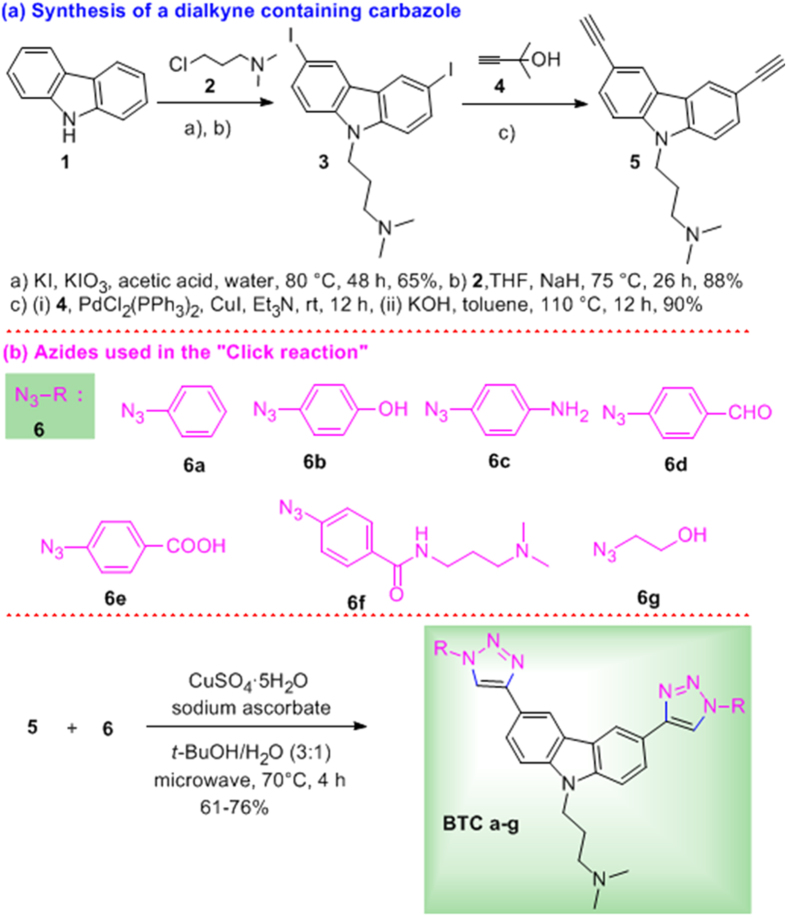
Modular synthesis of bis-triazolylcarbazole ligands (BTC).

For the synthesis, we prepared a carbazole dialkyne **5** from the commercially available carbazole **1**. The iodination^30^ of **1** followed by N-alkylation with 3-dimethylaminopropylchloride (**2**) afforded the diiodo compound **3**. Sonogashira coupling of **3** with 3-methyl butynol **4** followed by removal of the acetone group afforded the dialkyne **5** in 90% overall yield for the two steps. Carbazole dialkyne **5** was treated with the azides **6a–g** (see Supplementary Information) using catalytic CuSO_4_, sodium ascorbate in *t*-BuOH/H_2_O (3:1)^31^ under microwave conditions at 70 °C for 4 h to give the corresponding bis-triazolylcarbazole derivatives **BTC a-g** in high yields (Fig. 1 and Supplementary Chart S1).

### Bis-triazolylcarbazoles selectively stabilize G-quadruplex over duplex DNA

**Figure 2:**
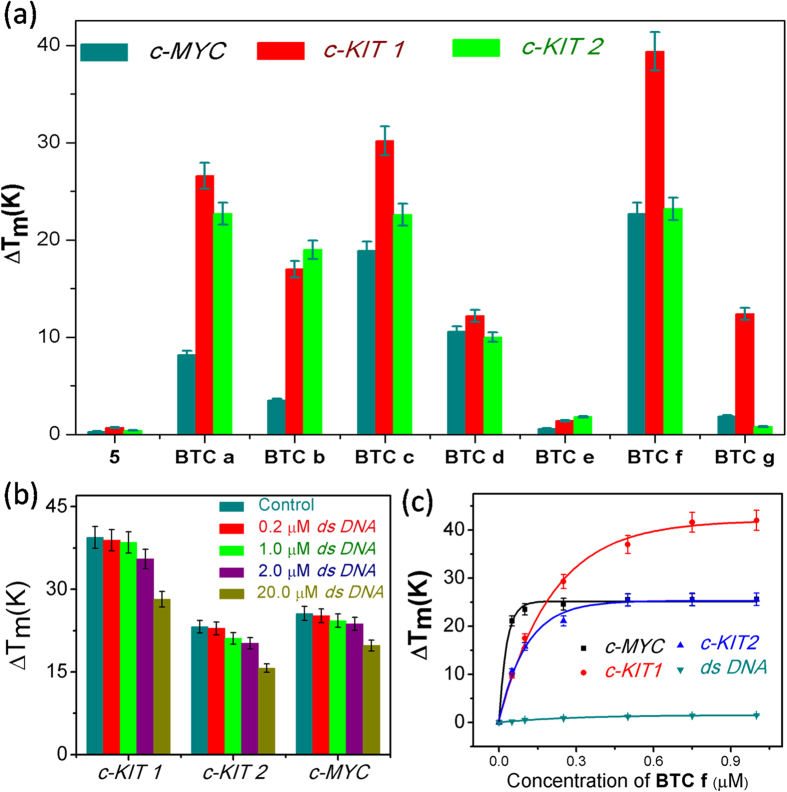
(**a**) FRET stabilization potential of carbazole derivatives **5** and **BTC a-g** (1.0 μM) upon binding to *c-MYC*, *c-KIT1* and *c-KIT2* quadruplex, T_m_ (°C) for *c-MYC* = (70.9 ± 1.2), *c-KIT1* = (53.0 ± 1.7), *c-KIT2* = (70.0 ± 1.4), *ds* DNA = (61.2 ± 2.1). (**b**) FRET competition assay of **BTC f** (1.0 μM) for G-quadruplexes (200 nM) in the presence of duplex DNA (200 nM, 1.0 μM, 2.0 μM and 20.0 μM). (**c**) Thermal shift profiles for **BTC f** upon stabilizing to quadruplexes and duplex DNA; buffer: 50 mM potassium cacodylate, pH 7.4.

Förster Resonance Energy Transfer (FRET) melting analysis^32^,^33^ was employed to determine the stabilization induced by carbazole derivatives (**5** and **BTC a–g**) by measuring the change in melting temperature (ΔT_m_) of the dual labeled (5′-FAM and 3′-TAMRA) G-rich oncogenic promoter sequences (*c-MYC, c-KIT1* and *c-KIT2*) and a control *ds DNA*. Ligand **BTC f** containing the three terminal–NMe_2_ groups exhibited maximum stabilization for quadruplexes at a ligand concentration of 1 μM. However, the starting dialkynecarbazole **5** hardly altered the T_m_ values of the quadruplexes. Ligands **BTC a-c** lacked the two cationic side chains and displayed moderate G-quadruplex stabilization. When the two cationic side chains of the carbazole were substituted by two anionic –COOH groups (ligand **BTC e**), a diminished stabilization for the quadruplex sequences was observed. These observations collectively suggest that changing the end substituents of the carbazole ligands has a profound impact on G-quadruplex stabilization (Fig. 2a and Supplementary Table S1). FRET competitive experiments were performed to evaluate the selectivity of **BTC f** for G-quadruplex over duplex DNA (Fig. 2b). FRET melting of 200 nM dual labeled *c-MYC, c-KIT1* and *c-KIT2* quadruplexes were performed with 1 μM **BTC f** in the presence of different concentrations of competitor *ds DNA* (0, 0.2, 1.0, 2.0, 20 μM). **BTC f** appeared to be highly selective towards the quadruplexes by maintaining high ΔT_m_ values for the quadruplexes even in the presence of 10 mol equivalent excess of *ds DNA*. At 100 mol equivalent excess of *ds DNA*, only a small change in ΔT_m_ values for the quadruplexes was observed. These results indicate that *ds DNA* does not significantly interfere with the binding of **BTC f** to the quadruplexes. Next, concentration-dependent FRET melting experiments of the promoter quadruplexes and *ds* DNA were performed. The melting profiles at various concentrations of **BTC f** demonstrate a dose-dependent increase in the values of ΔT_m_ for the quadruplexes (Fig. 2c and Supplementary Table S2). Ligand **BTC f** showed the highest Δ*T*_m_ for promoter gene quadruplexes that can be measured by this method. It showed a Δ*T*_m_ value of 22.7 ± 1.4 K (i.e. a *T*_m_ of 93 °C) for *c-MYC* at 100 nM, 39.4 ± 2.5 K (i.e. a *T*_m_ of 93 °C) for *c-KIT1* at 750 nM and 23.2 ± 1.6 K (i.e. a *T*_m_ of 93 °C) for *c-KIT2* at 500 nM ligand concentrations. These results revealed that 5–7 fold higher concentrations of **BTC f** are required for the *c-KIT1* and *c-KIT2* to achieve maximum stabilization potential (Δ*T*_m_) compared to the *c-MYC* quadruplex. To investigate whether **BTC f** could induce the formation of the G-quadruplex, FRET melting assay was performed in the absence of K^+^ ion. Interestingly, **BTC f** could stabilize G-quadruplex formation in the absence of any added K^+^ ion, pH 7.4 (Supplementary Fig. S1). Similar to the results of FRET melting in the presence of K^+^, lower concentrations (250 nM) of **BTC f** were required to obtain the highest Δ*T*_m_ for *c-MYC* in the absence of K^+^. Higher concentrations of **BTC f** are required for achieving maximum Δ*T*_m_ for the *c-KIT1* (1 μM) and *c-KIT2* (750 nM) quadruplexes. **BTC f** did not significantly change the melting temperature of *ds DNA* even at higher concentrations.

### BTC f as an environmentally sensitive “turn-on” probe for *c-MYC* G-quadruplex

**Figure 3:**
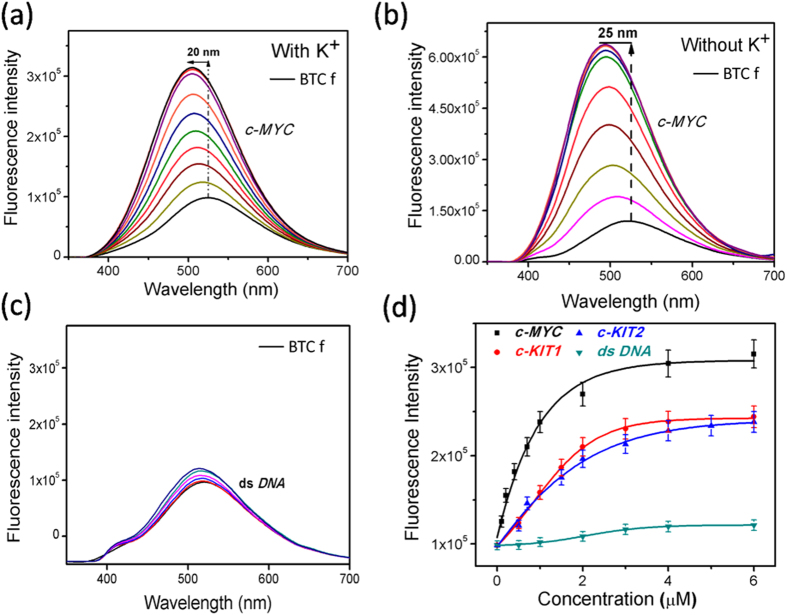
Fluorimetric titration spectra of **BTC f** (1.0 μM) with (0–6 eq) of (**a**) *c-MYC* in 100 mM Tris•HCl buffer at pH 7.4 containing 100 mM KCl, (**b**) *c-MYC* in 100 mM Tris•HCl buffer at pH 7.4 without additional KCl and (**c**) *ds DNA* in 100 mM Tris•HCl buffer at pH 7.4 containing 100 mM KCl. (**d**) Fluorescence intensity profiles of **BTC f** (1.0 μM) upon step-wise addition of quadruplex sequences (0–6 eq); titrations were performed in 100 mM Tris•HCl buffer at pH 7.4 containing 100 mM KCl.

The binding characteristics of **BTC f** with the G-quadruplex forming sequences (*c-MYC, c-KIT1* and *c-KIT2*) and duplex DNA (*ds DNA*) were investigated using fluorescence spectroscopy (Fig. 3 and Supplementary Fig. S2). The emission spectra of **BTC f** displayed a maxima at 522 nm (quantum yield, Φ = 0.054, abs, λ_max_ = 260, 290 nm), when excited at 290 nm in 100 mM Tris•HCl buffer, at pH 7.4 containing 100 mM KCl. Upon titration with the promoter quadruplexes (0–6 equiv.), **BTC f** (1.0 μM) showed an increase in fluorescence (2–3.3 fold) along with a blue shift (14–20 nm). The degree of fluorescence enhancement was the highest for *c-MYC* (3.3 fold) compared to the *c-KIT1* (2.0 fold) and *c-KIT2* (2.0 fold) quadruplexes. The blue shift was the largest for the *c-MYC* (20 nm) compared to the *c-KIT1* (15 nm) and *c-KIT2* (14 nm). Almost negligible fluorescence enhancement was observed (Fig. 3c) when **BTC f** was titrated with the *ds DNA* (0–6 equiv.). The dissociation constants (*K*_*D*_) calculated from the fluorescence spectra were found to be in the low micromolar range (0.3–1.38 μM). Ligand **BTC f** shows a 5-fold selectivity for *c-MYC* with a *K*_*D*_ value of (*K*_*D*_ = 300 ± 15 nM), over *c-KIT1* (*K*_*D*_ = 1.38 ± 0.07 μM) and *c-KIT2* (*K*_*D*_ = 1.37 ± 0.06 μM) quadruplexes. These results are consistent with the FRET melting data that **BTC f** shows selectivity for the *c-MYC* compared to the *c-KIT1* and *c-KIT2* quadruplexes. It is worth mentioning that **BTC f** exhibited a strong blue shift of 25 nm with a 5.5 fold increase in fluorescence upon titration with the *c-MYC* in the absence of K^+^ (Fig. 3b).

Next, the binding behaviour of **BTC c** with the *c-MYC* quadruplex was investigated (Supplementary Fig. S3a-b). The emission spectra of **BTC c** showed an emission maximum at 402 nm with a comparatively lower quantum yield (quantum yield, Φ = 0.044, abs, λ_max_ = 256, 342 nm) along with two weak shoulders, when excited at 330 nm in 100 mM KCl and 100 mM Tris•HCl buffer, at pH 7.4. When the fluorimetric titration of **BTC c** (1 μM) was carried out with increasing concentration of the *c-MYC* (0–6equiv.), only a slight increase in fluorescence intensity was observed. The difference in the fluorescence spectra between **BTC c** and **BTC f** may be attributed to the presence of an extended π-electron delocalization between the lone pair of electrons on triazole nitrogen atom of the donor and the acceptor carbonyl group in **BTC f**. These results indicate that **BTC f** is an effective light-up probe for the *c-MYC* quadruplex. In good agreement with the FRET melting results, **BTC f** that exhibited a higher stabilization potential showed several fold higher binding affinity for the *c-MYC* (*K*_*D*_ = 300 ± 15 nM) compared to **BTC c** (*K*_*D*_ > 20 μM).

In order to understand the observed blue shift upon interaction of **BTC f** with quadruplexes, the effect of environment polarity was analysed by recording the emission spectra of **BTC f** in various solvent environments such as ethyl acetate, DMF, DMSO, ethanol, methanol and water (Supplementary Fig. S4a). The observed emissions (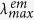) were plotted against Reichardt’s E_T_(30) polarity parameter^34^ (Supplementary Fig. S4b) to give a linear correlation (y = 4.03x + 267.3, r^2^ = 0.9292) over the range of the solvents. The Stokes shift (S) was calculated from the maximum wavelength (λ_max_) value of the excitation and emission spectra (Supplementary Table S4). Both 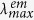 and S values showed their strong dependence on the polarity of the medium. We observed that the emission maximum of **BTC f** is blue-shifted from 522 to 410 nm and the fluorescence intensity is simultaneously enhanced as the solvent polarity decreases from water to ethylacetate. These observations suggest that the enhancement in the fluorescence intensity along with the blue-shift in the emission spectra of the **BTC f** with the quadruplexes is a consequence of the proximity of **BTC f** to the non-polar hydrophobic regions^35^ of the G-quadruplexes thereby manifesting the high affinity of **BTC f** towards the G-quadruplex structures.

### BTC acquires an induced CD signal upon interaction with *c-MYC* G-quadruplex

The circular dichroism (CD) spectrum of *c-MYC* quadruplex sequence showed a positive peak at 260 nm and a negative peak around 240 nm, in the absence of K^+^, which is characteristic of a parallel conformation^36^,^37^,^38^ (Supplementary Fig. S5). As potassium stabilizes G-quadruplex structure, the molar ellipticity of both positive and negative peaks of *c-MYC* was enhanced in K^+^ containing buffer (Supplementary Fig. S5). The incremental addition of **BTC f** to the *c-MYC* resulted in a slight decrease in the positive band at 260 nm in the presence and absence of K^+^. This suggests that **BTC f** binds to the parallel quadruplex structure of *c-MYC* and does not disrupt the structure. In addition, **BTC f** exhibited a positive induced circular dichroism (ICD) in the absorbing 290–350 nm range (centered at 322 nm) in the presence and absence of K^+^. It is worth mentioning that, no such ICD signal was detected upon binding of **BTC f** to *c-KIT 1* and *c-KIT 2* quadruplexes (Supplementary Fig. S5c-d), which indicates that **BTC f** can discriminate between the promoter quadruplexes.

UV/Vis spectroscopy indicated that **BTC f** exhibits hypochromism upon addition of the *c-MYC* (0–0.4 equiv.) and the peaks at 260–290 nm overlap with the *c-MYC* at higher concentration (Supplementary Fig. S6). The observed hypochromism in UV spectra suggests strong stacking interaction between aromatic chromophores of **BTC f** with the G-quadruplex base pairs and indicates possible binding of these ligands into the nonpolar site of the *c-MYC* quadruplex. The red shifted ICD band at 322 nm in CD spectra may arise due to the binding of the optically inactive **BTC f** inside the asymmetric microenvironment of the *c-MYC* quadruplex^39^. CD spectroscopic analysis further reveals that **BTC f** bound *c-MYC* structure is similar in the presence and absence of K^+^.

### Bis-triazolylcarbazoles selectively bind to a minor conformation of *c-MYC* quadruplex

**Figure 4:**
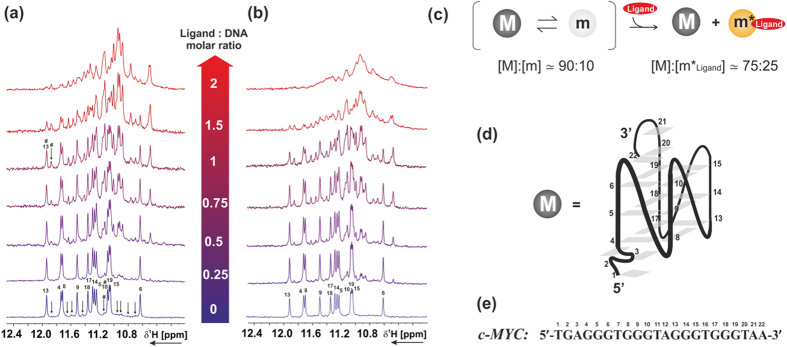
Imino region of 100 μM *c-MYC* in the presence of **BTC f** at different [ligand]:[DNA] molar ratio, (**a**) in 25 mM Tris·HCl buffer at pH 7.4 containing 100 mM KCl, 90% H_2_O/10% D_2_O (**b**) in 25 mM Tris·HCl buffer at pH 7.4 without additional KCl, 90% H_2_O/10% D_2_O. Titrations were performed at 298 K, 600 MHz. Signals from the major conformation of *c-MYC* are labeled according to the numbering shown in panel (**e**), while signals from the binding-competent minor conformation are marked with arrows. (**c**) Scheme of the proposed binding mechanism with estimated population of each state. Major conformation, minor conformation in the free-form and minor conformation in the ligand-bound form are indicated with M, m and m*_Ligand_, respectively. Population of the states in the absence of the ligand was estimated from the ratio of the integrals of signals marked in the spectrum with #, while population of the states in presence of ligand was estimated at a [ligand]:[DNA] molar ratio of 1, from the ratio of the integrals of signals marked in the spectrum with #. (**d**) Folding of the major conformation (M) of *c-MYC* determined by Ambrus *et al.*[PDB code: 1XAV]^13^. (**e**) *c-MYC* sequence used for NMR binding titration.

NMR titrations show that the ligand **BTC f** is strongly interacting with the *c-MYC* sequence, as suggested by the significant changes of the imino pattern profile upon addition of the ligand (Fig. 4). Figure 4a shows that upon addition of **BTC f**, a set of minor peaks (already observable in the DNA free form and indicated with arrows) becomes more intense, while the chemical shift and the line width of the imino protons of the major conformation are not perturbed. At a [ligand]:[DNA] molar ratio of 2, the intensity of the peaks of the major conformation is reduced, compared to the intensity of the peaks of the ligand-bound conformation. We propose that **BTC f** binds to the *c-MYC via* conformational selection: out of an ensemble of DNA conformers in equilibrium with each other (major conformation-Fig. 4d and one or several minor conformations), the ligand selects the minor-populated conformer and binds to it in a specific manner. Interestingly, **BTC f** binds to the minor conformer, most likely a parallel stranded structure with different capping structures^13^, and upon ligand binding, the equilibrium of the free form DNA^major^/DNA^minor^ conformational equilibrium is accordingly shifted. Moreover, upon ligand addition we also observe the appearance of new signals in the imino region (e.g., at 10.5 ppm) suggesting that the ligand **BTC f** can induce conformational changes in the structure of the binding-competent minor conformation. The proposed binding mechanism is summarized in Fig. 4c. The major conformation (M) is not binding-competent and the binding-competent minor conformation (m) undergoes small conformational changes upon ligand binding (m*_Ligand_). NMR titration of **BTC f** in the absence of KCl (Fig. 4b) shows that the ligand bound conformation of *c-MYC* resembles the one observed in the presence of 100 mM KCl. These results support FRET melting, fluorescence and CD studies that **BTC f** can stabilize the *c-MYC* quadruplex in the absence and presence of potassium. NMR titration of *c-MYC* with ligand **BTC c**, which lacks the two cationic side chains showed line broadening with no significant chemical shift perturbation of the imino proton signals of the DNA major conformation (Supplementary Fig. S7a). Similar to the [**BTC f**]:[*c-MYC*] complex, upon addition of **BTC c**, a new set of peaks was observed, which corresponds to one or more minor conformers of the *c-MYC* quadruplex. The NMR data suggest that these ligands interact with and stabilize a minor conformation of *c-MYC* quadruplex.

### Bis-triazolyl carbazoles inhibit the growth of cancer cells

To characterize the activity in biological systems, the antiproliferative activities of this class of compounds **BTC a–g** were determined in human hepatocellular liver carcinoma HepG2 cells using MTT assays^40^ (Supplementary Fig. S8 and Table S5). The results indicated that **BTC f** potently inhibits the growth of cancer cells at low micromolar concentrations with an IC_50_ value of ∼4.3 ± 0.69 μM. **BTC c** (IC_50_ = 11.38 ± 1.54 μM) displays a somewhat lower antiproliferative activity than **BTC f**. However, **BTC d-e** and **BTC g** do not show strong activity against HepG2 cells (Supplementary Table S5). We then evaluated the growth inhibitory activity of **BTC f** in two other human cancer cell lines such as breast carcinoma (MCF-7) and colon carcinoma (HCT 116), and a normal cell line (C2C12, mouse myoblast). **BTC f** exerts growth inhibition against human cancer cells, with IC_50_values of 6.8 ± 0.84 μM and 8.2 ± 1.2 μM for MCF 7 and HCT 116 cells respectively (Supplementary Fig. S8), but importantly it is less toxic to the model normal cell line (IC_50_ > 45 μM for C2C12, data not shown). It is worth noting that the main core carbazoledialkyne **5** does not show cytotoxicity towards normal as well as cancer cells (data not shown). These results indicate that the triazole ring and the side chains contribute towards the antiproliferative activities of the carbazole derivatives in cancer cells. Among the cancers cells (HepG2, MCF-7 and HCT 116), **BTC f** is the most potent in HepG2 cells. It has been reported that the expression *c-MYC* gene is elevated in liver carcinoma HepG2 cells compared to the normal human liver cells^2^. Biophysical analysis suggested that **BTC f** shows high specificity for the *c-MYC* G-quadruplex over duplex DNA. This encouraged us to evaluate the molecular mechanism underlying the anti-proliferative effect of **BTC f** in HepG2 cells. For a comparison, the effect of the less potent *c-MYC* quadruplex binding ligand **BTC c** with a reduced antiproliferative activity towards HepG2 cells was also investigated.

### Bis-triazolylcarbazoles down-regulate *c-MYC* expression

**Figure 5:**
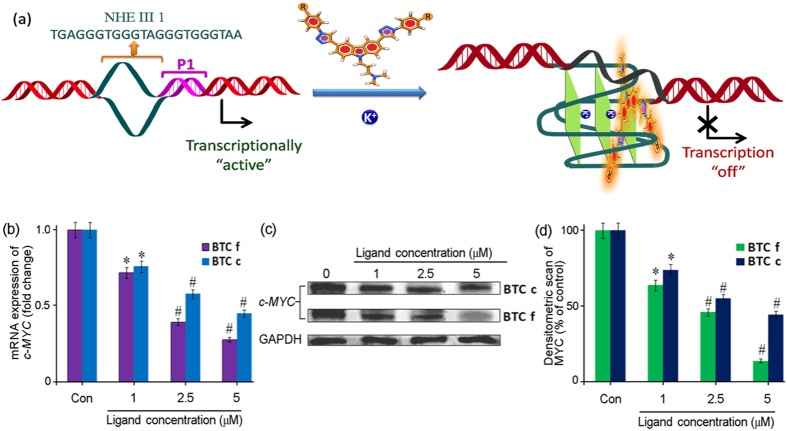
Effect of **BTC c** and **f** on the expression of *c-MYC* protein in human cancer cells : (**a**) Schematic representation of ligand induced transcriptional down-regulation of *c-MYC* gene. (**b**) Determination of transcriptional regulation of *c-MYC* mRNA in the presence of increasing concentration of **BTC c** or **BTC f** in cancer cells by qRT-PCR. qRT-PCR was performed and quantified by comparative threshold method using GAPDH as housekeeping gene. (**c**) Immunoreactive bands of MYC protein were analyzed by Western blot. (**d**) Densitometric analyses of immunoblots showing concentration dependent reduced level of MYC protein. Results are representative of five independent experiments with two replicates. The data are shown as mean ± SD. *P < 0.05, #*P* < 0.01, versus untreated cancer cells.

Ligands **BTC c** and **BTC f** were evaluated for their effect on transcriptional regulation of *c-MYC* in HepG2 cells (Fig. 5a). Total mRNA was isolated from HepG2 cells after treatment with varying concentrations (1.0, 2.5 and 5.0 μM) of **BTC c** and **BTC f** for 24 h. The level of *c-MYC* mRNA was quantified using quantitative real-time polymerase chain reaction (qRT-PCR) and the gene expression was normalized relative to the expression of a constitutively expressed house-keeping gene, glyceraldehyde-3-phosphate dehydrogenase (GAPDH). Analysis of the qRT-PCR data revealed that both **BTC c** and **BTC f** are able to reduce the level of *c-MYC* mRNA in a dose dependent manner (Fig. 5b). At 5.0 μM concentration, **BTC f** reduces the level of *c-MYC* mRNA by 75 ± 3% relative to the control. Compared to that, **BTC c** at 5.0 μM leads to a suppression of *c-MYC* mRNA level by 48 ± 3% of the control suggesting its relatively lower efficacy than **BTC f**. The GAPDH mRNA is equally expressed in control, **BTC c** and **BTC f** treated HepG2 cells, which confirms that the reduction of mRNA level due to **BTC c** and **BTC f** is *c-MYC* gene-specific. To investigate that the observed reduction in *c-MYC* mRNA levels leads to an inhibition of MYC protein, we have employed western blotting using anti-MYC antibody (Fig. 5c,d). Protein levels were measured for the *c-MYC* and the housekeeping gene GAPDH in HepG2 cells after treatment with **BTC c** and **BTC f** for 24 h at final concentrations of 1.0, 2.5 and 5.0 μM. Both **BTC c** and **BTC f** exhibit reduced expression of MYC protein in a dose dependent manner compared to the untreated HepG2 cells (Fig. 5c,d), which is in good agreement with the qRT-PCR analysis data. The densitometric analysis of the blots revealed that the decrease of MYC expression in cancer cells is in the range of 85 ± 3% and 52 ± 2% at 5.0 μM concentration of **BTC f** and **BTC c**, respectively. The concentration required for 50% inhibition in *c-MYC* expression 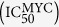 is approximately 2 μM for **BTC f** and 4.6 μM for **BTC c**. Almost negligible reduction in GAPDH expression was observed in both treated and untreated cells. Together these results suggest that the **BTC** ligands can reduce the expression of MYC protein in HepG2 cancer cells at mRNA and protein levels (Fig. 5a).

### BTC f inhibits cell cycle progression and induces apoptosis

**Figure 6:**
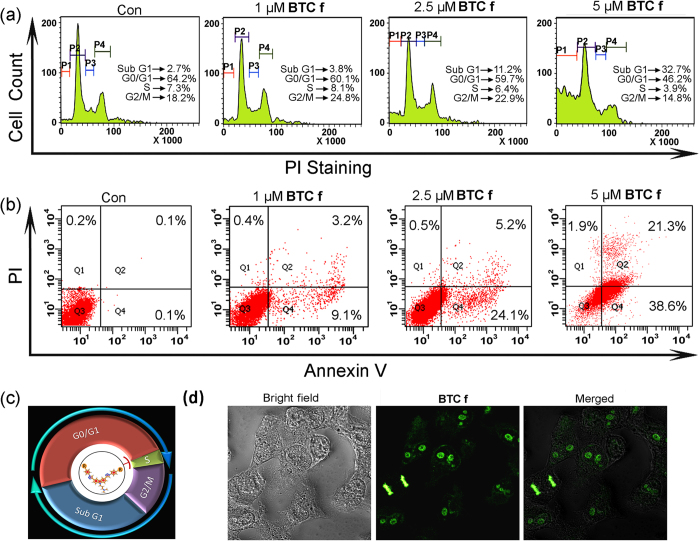
(**a**) Flow cytometric analysis of cell cycle parameters after incubation of **BTC f** (1.0–5.0 μM) in HepG2 cancer cells. P1, P2, P3 and P4 represent cell population at Sub G1, G0/G1, S and G2/M respectively. (**b**) Flow cytometric analysis of the mode of cancer cell death after treatment with **BTC f** (1.0–5.0 μM) in HepG2 cancer cells; Lower left (Q3), lower right (Q4), upper right (Q2) and upper left (Q1) quadrants indicate healthy cells, early, late apoptotic and necrotic cells, respectively. (**c**) Schematic representation of cell cycle arrest at Sub G1 phase by **BTC f**. (**d**) Confocal Microscope images (400 X magnification) showing localization of **BTC f**. All results are representative of three independent experiments with similar results.

To determine whether **BTC f** mediated inhibition of HepG2 cell proliferation was associated with cell cycle arrest, we performed cell cycle analysis by propidiumiodide (PI) staining using flow cytometer. Flow cytometry analysis of **BTC f** (1.0–5.0 μM) treated HepG2 cells showed an increase in the SubG1 population (2.7% to 32.7%) with the increasing concentration of **BTC f** (Fig. 6a). Interestingly, at lower concentrations of **BTC f**, the cell population in G2 phase was increased (18.2% to 24.8%), and as the concentration of **BTC f** is increased, both S and G2 phase populations were decreased (7.3% to 3.9% and 18.2% to 14.8%, respectively) with a subsequent increase in the population of SubG1 phase.

To gain insight into the mechanism by which **BTC f** induced cell death of HepG2 cells, flow cytometry was employed to investigate the mode of cell death using Annexin-V and PI dual staining assay (Fig. 6b). HepG2 cells were incubated with **BTC f** (1.0–5.0 μM) for 24 h and the untreated cells were used as control. The flow cytometry analysis revealed that the apoptotic cell population was increased significantly (0.2% to 59.9%) in a dose-dependent manner. However, the extent of necrotic death was only 1.9% under the experimental conditions. This is in well agreement with the reduced activity of *c-MYC* in liver carcinoma cells after treatment with **BTC f**.

### BTC f as a fluorescent probe for live-cell nucleus imaging

Confocal laser scanning microscopy (CLSM) was employed to examine the cellular localization of **BTC f** in living cells. HepG2 cells were treated with **BTC f** (5.0 μM) for 4 h and the CLSM images were taken, which clearly showed that the **BTC f** selectively stains the nucleus compared to the cytoplasm. The merged image (Fig. 6d) establishes the localization of **BTC f** within the nucleus. These data suggest that **BTC f** is cell-permeable and binds to the cellular DNA. As **BTC f** shows higher affinity towards the G-quadruplex over duplex DNA, its localization in the nucleus area indicates that **BTC f** may induce apoptosis by stabilizing the G-quadruplex in HepG2 cells.

## Discussion

**Figure 7:**
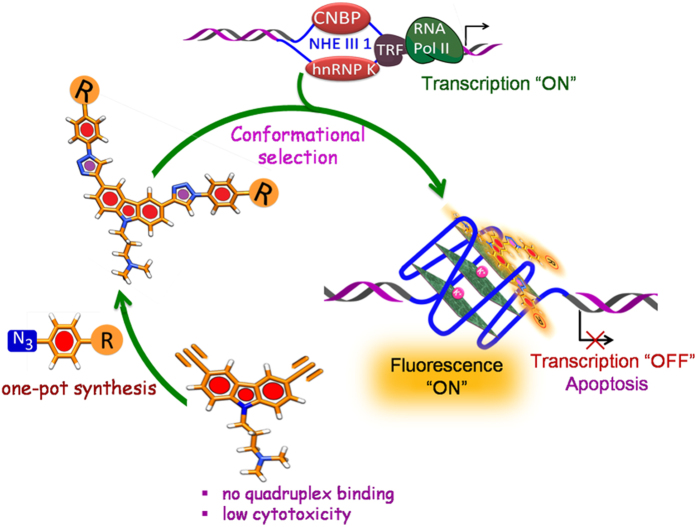
Bis-triazolylcarbazole derivatives stabilize *c-MYC* G-quadruplex and inhibit *c-MYC* transcription.

A modular access to bis-triazolyl carbazole derivatives has been devised, where a simple carbazole dialkyne precursor **5** was prepared from the commercially available carbazole. The carbazole dialkyne **5** was treated with a variety of azides **6a**-**g** using Cu(I) catalyzed azide-alkyne cycloaddition to preapre the corresponding bis-triazoloylcarbazole derivatives **BTC a-g** in high yields (Fig. 1). FRET melting analysis (Fig. 2) revealed that the carbzoledialkyne **5** does not change the melting temperature of any of the three investigated promoter quadruplexes (*c-MYC*, *c-KIT1* and *c-KIT2*) or the duplex DNA, while the bis-triazoloyl carbazole derivative **BTC f** with two carboxamide side chains exhibits high stabilization potential for the quadruplexes over the duplex DNA (Fig. 2 and Fig. 7). The diamino bis-triazolylcarbazole **BTC c** displayed moderate stabilization for the G-quadruplex sequences. Competitive FRET-melting experiments (Fig. 2b) clearly showed that the presence of 100 mol equivalent excess *ds* DNA did not significantly interfere with the stabilization of quadruplexes induced by the ligand **BTC f**, indicating its high selectivity for the quadruplexes over duplex DNA. FRET melting experiments at various concentrations of **BTC f** revealed that higher concentrations (5–7 fold) of **BTC f** are required to achieve the maximum stabilization potential (Δ*T*_m_) for *c-KIT1* and *c-KIT2* quadruplexes compared to the *c-MYC* quadruplex. These results indicated that **BTC f** shows a preference for the *c-MYC* over the *c-KIT1* and *c-KIT2* quadruplexes. **BTC f** can also stabilize the quadruplexes to attain the maximum stabilization potential in the absence of K^+^ ion. Despite showing high stabilization potential for the quadruplexes, **BTC f** did not exhibit any detectable stabilisation for the *ds DNA*.

Fluorescence studies showed that **BTC f** is a microenvironment-sensitive fluorescence “turn-on” sensor that selectively detects the *c-MYC* quadruplex over the duplex DNA with an enhancement in the fluorescence intensity with a blue shift (Fig. 3). However, the carbazole derivative **BTC c** in the presence of *c-MYC* neither triggered a significant fluorescence “turn-on” response nor a blue shift. The difference in fluorescence properties between **BTC c** and **BTC f** may be attributed to the lack of extended π-electron delocalization in **BTC c**. In agreement with the FRET melting results, the fluorescence spectroscopy studies showed high specificity of **BTC f** for the quadruplexes over duplex DNA, and in particular, **BTC f** showed a 5-fold preference for *c-MYC* (*K*_*D*_ = 300 nM) over *c-KIT1* and *c-KIT2* promoter quadruplexes. Moreover, the confocal laser scanning microscopy (CLSM) shows that **BTC f** can be successfully applied to visualize the live-cell nucleus of HepG2 cells with high selectivity (Fig. 6).

NMR and Circular Dichroism (CD) spectroscopic analyses were employed to gain insight into the structural basis for the recognition of **BTC c** and **BTC f** to *c-MYC* quadruplex. CD binding titrations revealed that the binding of **BTC f** to *c-MYC* gives rise to a CD signal (ICD peak) from the bound ligand (Supplementary Fig. S5a-b). No such ICD bands are observed upon interaction of **BTC f** with the *c-KIT1* and *c-KIT2* quadruplexes, which indicates that **BTC f** binds to the *c-MYC* in an asymmetric fashion. It has been reported that multiple conformations of 3′- and 5′-flanking capping structures of *c-MYC* quadruplex co-exist at equilibrium^13^. NMR analyses (Fig. 4) suggested that **BTC f** selects and binds specifically to one of these minor conformations of the *c-MYC via* conformational selection. The [**BTC f**]:[*c-MYC*] interaction process is similar with or without K^+^. The binding of **BTC f** could also induce a conformational change in the binding-competent minor conformation of the *c-MYC*, which may be useful to specifically alter the biological function of the *c-MYC*.

Among the synthesized carbazole analogues, compound **BTC f** was found to be the most potent molecule that inhibited the growth of human hepatocellular liver carcinoma HepG2 cells (which highly expresses MYC) at a low micromolar concentration (IC_50_ = 4.3 ± 0.69 μM) without affecting the normal mouse myoblast C2C12 cells. The less potent quadruplex stabilizer **BTC c** also showed good antiproliferative activity with an IC_50_ value of 11.38 ± 1.54 μM in HepG2 cells. It is worth mentioning that the starting carbazoledialkyne **5** neither stabilized the G-quadruplex DNA (FRET melting data) nor effectively inhibited the growth of cancer cells (Fig. 7). Since **BTC f** exhibits high specificity for the *c-MYC* quadruplex and the *c-MYC* gene is overexpressed in liver carcinoma HepG2 cells, the effect of **BTC f** and the less potent **BTC c** on transcriptional regulation *c-MYC* gene was studied in HepG2 cells (Fig. 5). **BTC f** was able to inhibit *c-MYC* expression in both transcriptional and translational level as suggested by qRT-PCR and Western blotting analysis (Fig. 5). Analysis of the cell cycle data revealed that cells were unable to transit from G1 phase to S phase with increasing concentration of **BTC f.** The increase in concentration of **BTC f** triggered cell cycle arrest in SubG1 phase and subsequent apoptosis in cancer cells, probably by down-regulating *c-MYC* gene expression^41^,^42^,^43^ as suggested by qRT-PCR and Western blot analysis (Figs 5 and 6). Our results collectively suggest that the reduction in *c-MYC* expression is probably due to the binding of **BTC f** to the *c-MYC* promoter-quadruplex. However, the exact molecular mechanism of **BTC f** mediated *c-MYC* down-regulation^44^ is currently under investigation.

This work highlights that a simple synthetic protocol can be devised to synthesize fluorescent bis-triazolylcarbazole derivatives, which can effectively inhibit *c-MYC* transcriptional activity (Fig. 7). The carbazole derivative with carboxamide side chains shows pronounced environment-sensitive fluorescence and selectively detects the *c-MYC* G-quadruplex over the duplex DNA via “turn-on” fluorescence. The ligand binds to the *c-MYC* with nM binding affinity and shows a five-fold preference for the *c-MYC* over the *c-KIT* promoter quadruplexes. The ligand can bind and stabilize the *c-MYC* quadruplex in the presence and absence of added K^+^. It is intriguing that the ligand can be used to ‘programme’ the *c-MYC* to adopt a specific conformation, which is stable with or without K^+^. The specific nucleic acid structure may find applications in the fields of nanobiotechnology and biomedical technology. This small molecule probe is an attractive probe for bio-imaging as it can rapidly and selectively stain the nucleus in living cells. Further this small molecule can induce cell cycle arrest and promote cancer cell death by apoptosis. These results collectively suggest that the carbazole derivative is a potent anticancer agent and a viable lead for further development of anticancer drugs.

### Synthetic Protocols

#### Synthesis of 3-(3,6-diiodo-9H-carbazol-9-yl)-N,N-dimethylpropan-1-amine 3

A mixture of carbazole **1** (15.0 g, 89.82 mmol), KI (19.36 g, 116.62 mmol), KIO_3_ (19.20 g, 89.82 mmol) in acetic acid (100 mL) and deionized water (10 mL) was stirred at 80 °C for 48 h under N_2_ atmosphere. After cooling to room temperature, the mixture was filtered, washed with deionized water and saturated Na_2_CO_3_ solution to afford 3, 6-diiodocarbazole as a colorless solid (24.4 g, 65%). ^1^H NMR (500 MHz, CDCl_3_): 8.32 (s, 2H), 8.09 (s_br_, 1H), 7.68 (d, 2H, *J* = 8.4 Hz), 7.21 (d, 2H, *J* = 8.4 Hz); ^13^C NMR (100 MHz, CDCl_3_): 138.5, 134.8, 129.4, 124.6, 112.7, 82.4.

A mixture of 3, 6-diiodocarbazole (500 mg, 1.193 mmol) and NaH (114.24 mg, 4.77 mmol) in 20 mL THF was stirred at room temperature for 2 h under nitrogen atmosphere. In another flask, 3-dimethylaminopropyl chloride hydrochloride **2** (435.33 mg, 3.58 mmol) and NaOH (1.0 g) were diluted in 5 mL water and cooled to room temperature, then the upper layer of this solution was added drop-wise to the above mixture. The reaction mixture was refluxed for an additional 24 h. After the removal of the solvent under reduced pressure, the residue was purified by column chromatography on silica gel using DCM/methanol (20:1) as eluent. Re-crystallization from ethanol gave the compound **3** (529 mg, 88%) as a colorless solid, mp 129–131 °C. ^1^H NMR (500 MHz, CDCl_3_): 8.32 (s, 2H), 7.70 (d, 2H, *J* = 8.4 Hz), 7.25 (d, 2H, *J* = 9.2 Hz), 4.32 (t, 2H, *J* = 6.7 Hz), 2.20 (s, 8H), 1.96 (t, 2H, *J* = 6.7 Hz); ^13^C NMR (100 MHz, CDCl_3_): 139.6, 134.5, 129.3, 124.0, 111.0, 81.8, 56.1, 45.2, 40.6, 26.5. HRMS (ESI) calculated for [C_17_H_18_I_2_N_2_]: 504.9632, Found 504.9635.

#### Synthesis of 3-(3,6-diethynyl-9H-carbazol-9-yl)-N,-N-dimethylpropan-1-amine 5

To a solution of **2** (300 mg, 0.595 mmol) in Et_3_N (5 mL) added PdCl_2_(PPh_3_)_2_ (35.1 mg, 0.05 mmol) and CuI (19.04 mg, 0.1 mmol) at room temperature. The mixture was stirred for 30 min and then 2-methylbut-3-yn-2-ol **4** (0.182 mL, 1.785 mmol) was added drop-wise. The resulting mixture was stirred under an argon atmosphere for 12 h, concentrated, washed with brine and dried over anhydrous Na_2_SO_4_. The crude product was purified by column chromatography. Then the resulting crude alcohol was refluxed with 5 equiv. KOH in toluene under an argon atmosphere for 12 h. The reaction mixture was concentrated, washed with brine and dried over anhydrous Na_2_SO_4_. The crude product was purified by column chromatography to give the dialkyne **5** (161 mg, 90% yield) as a yellow liquid. ^1^H NMR (500 MHz, CDCl_3_): 8.22 (s, 2H), 7.60 (d, 2H, *J* = 8.4 Hz), 7.41 (d, 2H, *J* = 9.3 Hz), 4.38 (t, 2H, *J* = 6.7 Hz), 3.08 (s, 2H), 2.22 (s, 8H), 1.99 (t, 2H, *J* = 6.7 Hz); ^13^C NMR (100 MHz, CDCl_3_): 141.0, 130.4, 124.9, 122.5, 113.0, 109.4, 85.0, 75.7, 56.4, 45.6, 41.0, 27.0; IR (KBr, cm^−1^): 3284, 3269, 2925, 2817, 2767, 2100, 1627, 1595, 1481, 1344, 1286, 1249, 1211, 1134; HRMS (ESI) calculated for [C_21_H_21_N_2_]: 301.1699, Found 301.1696.

#### General procedure for the synthesis of bis-triazole derivatives **BTC a-g** using click chemistry (GP1)

Dialkyne **5** (50 mg, 0.166 mmol) was dissolved in a 1:2 mixture of *t*-BuOH/H_2_O (4 mL). Copper(II) sulphate pentahydrate (4.14 mg, 0.0166 mmol) and sodium ascorbate (3.2 mg, 0.0166 mmol) were added and the solution was stirred for 10 min. The corresponding azide **6** (2.5 × 0.166 mol equiv.) was added and the mixture was then heated for 4 h at 70 °C under microwave irradiation. After cooling to room temperature, the reaction mixture was concentrated. The crude product was purified by flash column chromatography (from CH_2_Cl_2_ (100%) to CH_2_Cl_2_/MeOH (10:1) to CH_2_Cl_2_/MeOH/NH_4_OH (10:1:0.5) to give the corresponding bis-traizole derivatives ****BTC a-g****.

#### Synthesis of **BTC a**

Following the **GP-1**, the reaction of the dialkyne **5** with azide **6a** (49 mg) afforded **BTC a** (55 mg, 61%) as a yellow viscous liquid. ^1^H NMR (500 MHz, DMSO-d_6_): 9.35 (s, 2H), 8.84 (s, 2H), 8.10 (d, 2H, *J* = 8.8 Hz), 8.00 (d, 4H, *J* = 7.8 Hz), 7.78 (d, 2H, *J* = 8.8 Hz), 7.66 (t, 4H, *J* = 6.4 Hz), 7.53 (t, 2H, *J* = 6.8 Hz), 4.50 (t, 2H, *J* = 7.3 Hz), 2.26 (t, 2H, *J* = 5.8 Hz), 2.17 (s, 6H), 1.98 (t, 2H, *J* = 6.8 Hz); ^13^C NMR: (125 MHz, DMSO-d_6_): 148.3, 140.4, 136.8, 129.9, 128.5, 123.8, 122.4, 121.5, 119.8, 118.6, 117.3, 110.0, 55.8, 44.9, 40.2 (merged with DMSO-d_6_), 22.0; IR (KBr, cm^−1^): 3342, 3303, 2960, 2852, 1641, 1629, 1591, 1514, 1481, 1197, 1033, 1001; HRMS (ESI) calculated for [C_33_H_31_N_8_]: 539.2672, Found 539.2664.

#### Synthesis of *BTC b*

Following the **GP-1**, the reaction of the dialkyne **5** with azide **6b** (56 mg) afforded **BTC b** (70 mg, 74%) as a deep brown solid, mp > 220 °C; ^1^H NMR (500 MHz, DMSO-d_6_): 10.02 (s, 2H), 9.16 (s, 2H), 8.81 (s, 2H), 8.07 (d, 2H, *J* = 8.4 Hz), 7.74-7.77 (m, 6H), 7.00 (d, 4H, *J* = 8.4 Hz), 4.49 (t, 2H, *J* = 6.7 Hz), 2.32 (t, 2H, *J* = 6.7 Hz), 2.20 (s, 6H), 1.99 (t, 2H, *J* = 6.7 Hz); ^13^C NMR: (125 MHz, DMSO-d_6_): 157.7, 148.0, 140.3, 128.9, 123.7, 122.4, 121.7, 118.5, 117.2, 116.1, 109.9, 55.8, 44.7, 40.8 (merged with DMSO-d_6_), 26.2; IR(KBr, cm^−1^): 3126, 3026, 2952, 2831, 2790, 1604, 1521, 1481, 1465, 1452, 1282, 1220, 1164, 1058, 995; HRMS (ESI) calculated for [C_33_H_31_N_8_O_2_^+^]: 571.2564, Found 571.2567.

#### Synthesis of **BTC c**

Following the **GP-1**, the reaction of the dialkyne **5** with azide **6c** (56 mg) afforded **BTC c** (65 mg, 68.5%), a deep yellow solid, mp 110-112 °C; ^1^H NMR (500 MHz, DMSO-d_6_): 9.04 (s, 2H), 8.79 (s, 2H), 8.06 (dd, 2H, *J* = 8.5, 1.2 Hz), 7.73 (d, 2H, *J* = 8.2 Hz), 7.58 (d, 4H, *J* = 8.8 Hz), 6.74 (d, 4H, *J* = 8.8 Hz), 5.52 (s, 4H), 4.48 (t, 2H, *J* = 6.3 Hz), 2.22 (t, 2H, *J* = 6.9 Hz), 2.14 (s, 6H), 1.96 (t, 2H, *J* = 6.9 Hz); ^13^C NMR: (125 MHz, DMSO-d_6_): 149.3, 147.8, 140.3, 126.2, 123.7, 122.5, 121.9, 121.4, 118.2, 117.2, 113.9, 109.9, 55.9, 44.8, 40.8 (merged with DMSO-d_6_) 26.4; IR (KBr, cm^−1^): 3338, 3134, 2702, 2441, 1623, 1521, 1477, 1220, 1043; HRMS (ESI) calculated for [C_33_H_32_N_10_]: 569.2884, Found 569.2886.

#### Synthesis of **BTC d**

Following the **GP-1**, the reaction of the dialkyne **5** with azide **6d** (61 mg) afforded **BTC d** (65 mg, 66%) as an orange solid, mp > 220 °C; ^1^H NMR (500 MHz, DMSO-d_6_): 10.10 (s, 2H), 9.52 (s, 2H), 8.85 (s, 2H), 8.26 (d, 4H, *J* = 8.5 Hz), 8.19 (d, 4H, *J* = 8.6 Hz), 8.10 (d, 2H, *J* = 8.6 Hz), 7.80 (d, 2H, *J* = 8.6 Hz), 4.51 (t, 2H, *J* = 6.1 Hz), 2.36 (s, 2H), 2.23 (s, 6H), 2.01 (t, 2H, *J* = 6.1 Hz); ^13^C NMR: (125 MHz, DMSO-d_6_): 192.1, 148.7, 140.6, 135.6, 131.3, 123.9, 122.5, 121.2, 119.9, 118.7, 117.4, 112.9, 110.1, 55.7, 43.6, 40.8 (merged with DMSO-d_6_), 26.1; IR (KBr, cm^−1^): 2954, 2852, 1697, 1602, 1481, 1406, 1307, 1209, 1163, 1033; HRMS (ESI) calculated for [C_35_H_31_N_8_O_2_]: 595.2564, Found 595.3433.

#### Synthesis of **BTC e**

Following the **GP-1**, the reaction of the dialkyne **5** with azide **6e** (68 mg) afforded **BTC e** (70 mg, 67%) as a light yellow solid, mp > 220 °C; ^1^H NMR (500 MHz, DMSO-d_6_): 10.71 (s, 2H), 9.49 (s, 2H), 8.85 (s, 2H), 8.21–8.10 (m, 10H), 7.86 (d, 2H, *J* = 8.4 Hz), 4.59 (s, 2H), 3.20 (s, 2H), 2.73 (s, 6H), 2.50 (s, 2H); ^13^C NMR: (125 MHz, DMSO-d_6_): 166.4, 148.6, 140.3, 139.6, 131.1, 130.5, 123.9, 122.6, 121.6, 119.5, 118.7, 117.4, 110.2, 54.2, 48.5, 39.7 (merged with DMSO-d_6_), 23.7; IR (KBr, cm^−1^): 3406, 3126, 2628, 2113, 1703, 1606, 1481, 1382, 1224, 1039; HRMS (ESI) calculated for [C_35_H_31_N_8_O_4_]: 627.2463, Found 627.2465.

#### Synthesis of **BTC f**

Following the **GP-1**, the reaction of the dialkyne **5** with azide **6f** (102 mg) afforded **BTC f** (96 mg, 73%) as a yellow solid, mp 69-71 °C; ^1^H NMR (500 MHz, DMSO-d_6_): 9.43 (s, 2H), 8.85 (s, 2H), 8.70 (t, 2H, *J* = 5.9 Hz), 8.11 (s, 8H), 8.10 (s, 2H), 7.79 (d, 2H, *J* = 8.4 Hz), 4.51 (t, 2H, *J* = 5.9 Hz), 3.32 (4H, merged with water peak), 2.29 (t, 4H, *J* = 6.7 Hz), 2.22 (t, 2H, *J* = 6.7 Hz), 2.16 (s, 18H), 1.98 (t, 2H, *J* = 6.7 Hz), 1.67–1.72 (m, 4H); ^13^C NMR: (125 MHz, DMSO-d_6_): 165.1, 148.6, 140.6, 138.4, 134.4, 129.0, 122.5, 121.4, 119.4, 118.6, 117.4, 110.1, 56.9, 56.0, 43.6, 40.9 (merged with DMSO-d_6_), 38.9, 27.0, 26.5; IR (KBr, cm^−1^): 3380, 3062, 2657, 2432, 1645, 1608, 1548, 1508, 1481, 1296, 1224, 1037; HRMS (ESI) calculated for [C_45_H_55_N_12_O_2_]: 795.4565, Found 795.4778.

#### Synthesis of **BTC g**

Following the **GP-1**, the reaction of dialkyne **5** with azide **6g** (36 mg) afforded **BTC g** (60 mg, 76%) as a brown liquid; ^1^H NMR (500 MHz, DMSO-d_6_): 8.72 (s, 2H), 8.56 (s, 2H), 7.98 (dd, 2H, *J* = 8.5, 1.5 Hz), 7.68 (d, 2H, *J* = 8.5 Hz), 5.16 (s, 2H), 4.49–4.43 (m, 6H), 3.87 (t, 4H, *J* = 4.9 Hz), 2.20–2.10 (m, 8H), 1.94 (t, 2H, *J* = 6.7 Hz); ^13^C NMR: (125 MHz, DMSO-d_6_): 147.1, 140.1, 123.5, 122.4, 122.2, 120.9, 117.1, 109.8, 59.9, 55.9, 52.4, 45.1, 40.8 (merged with DMSO-d_6_), 27.8; IR (KBr, cm^−1^): 3271, 2667, 1600, 1481, 1465, 1244, 1135, 887; HRMS (ESI) calculated for [C_25_H_31_N_8_O_2_]: 475.2564, Found 475.2576.

### Online Methods

#### FRET melting experiments

Stock solutions having 1 μM concentration of each carbazole compound was prepared in MQ water (**BTC f**) and DMSO (compound **5**, **BTC a**–**e** and **BTC g**). Four dual fluorescently labeled DNA oligonucleotide sequences were diluted in 50 mM potassium cacodylate buffer, pH 7.4.

*c-MYC*: 5′-*FAM*-d(TGAG_3_TG_3_TAG_3_TG_3_TA_2_)-*TAMRA*-3′

*c-KIT1*: 5′-*FAM*-d(GGGAGGGCGCTGGGAGGGAGGG)-*TAMRA*-3′,

*c-KIT2*: 5′-*FAM-*d(GGGCGGGCGCGAGGGAGGGG)-*TAMRA-*3′ and

*ds DNA*: 5′-*FAM*-d(CCAGTTCGTAGTAACCC)-3′/3′-*TAMRA* (GGTCAAGCATCATTGGG)-5′

The donor fluorophore was 6-carboxyfluorescein, FAM, and the acceptor fluorophore was 6-carboxytetramethylrhodamine, TAMRA. Dual-labeled DNA was annealed at a concentration of 200 nM by heating at 95 °C for 5 min followed by cooling to room temperature. The 96-well plates were prepared by aliquoting 50 *μ*L of the annealed DNA into each well, followed by 50 *μ*L of the carbazole compounds.

For competition experiments, duplex competitors were added to 200 nM quadruplex sequences at final concentration of 200 nM, 1.0 *μ*M, 2.0 *μ*M and 20 *μ*M. The concentration of **BTC f** was kept at 1.0 *μ*M. For FRET titration experiments different concentration of **BTC f** (0.05, 0.1, 0.2, 0.4, 0.6, 0.8, 1.0 *μ*M) were added to 100 nM of all four DNA sequences. Measurements were made in triplicate with an excitation wavelength of 483 nm and a detection wavelength of 533 nm using a LightCycler® 480-II System RT-PCR machine (Roche). Final analysis of the data was carried out using Origin Pro 8 data analysis.

#### Fluorimetric titration

Fluorescence emission spectra were recorded with successive addition of the quadruplex solution into the ligand solution. The fluorescence spectra were recorded on a Horiba JobinYvonFluoromax 3 instrument at 25 °C in a thermostated cell holder using quartz cuvette with a 1 cm path-length. The spectra were taken using filtered and degassed buffer (100 mM Tris-HCl containing 100 mM KCl) and other solvents like ethyl acetate, DMF, DMSO, ethanol, methanol and water. In this experiment, 1.0 μM **BTC f** was titrated with aliquots of different preannealed DNA sequences:

*c-MYC*: 5′-d(TGAG_3_TG_3_TAG_3_TG_3_TA_2_)-3′

*c-KIT1*: 5′-d(GGGAGGGCGCTGGGAGGGAGGG)-3′,

*c-KIT2*: 5′-d(GGGCGGGCGCGAGGGAGGGG)*-*3′ and

*ds DNA*: 5′-d(CCAGTTCGTAGTAACCC)-3′/3′*-*(GGTCAAGCATCATTGGG)-5′

The recorded spectral data was used to determine the dissociation constant of the ligands for quadruplexes using the Hill-1 formula (eqn. 1):
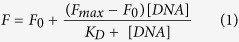
*F* is the fluorescence intensity, *F*_*max*_ is the maximum fluorescence intensity, *F*_*0*_ is the fluorescence intensity in the absence of DNA and *K*_*D*_ is the dissociation constant.

#### CD spectroscopy

CD spectra were recorded on a JASCO J-815 spectrophotometer by using a 1 mm path length quartz cuvette. Aliquots of **BTC f** were added stepwise to pre-annealed *c-MYC* (TGAG_3_TG_3_TAG_3_TG_3_TA_2_), *c-KIT1* (GGGAGGGCGCTGGGAGGGAGGG) and *c-KIT2* (GGGCGGGCGCGAGGGAGGGG) quadruplex sequences in Tris•HCl (100 mM) buffer at pH 7.4 containing KCl (100 mM). The CD spectra were also recorded upon incremental addition of **BTC f** to the *c-MYC* quadruplex sequence in Tris•HCl buffer (100 mM), at pH 7.4 in the absence of KCl. The DNA concentrations used were 10 μM. The CD spectra represent an average of three scans and were smoothed and zero corrected. Final analysis and manipulation of the data was carried out by using Origin 8.0.

#### NMR spectroscopy

NMR experiments were performed using intramolecular G-quadruplex formed by *c-MYC* purchased by Eurofins MWG Operon (Ebersberg, Germany) as HPSF^**®**^ (High Purity Salt Free) purified oligos and further purified *via* HPLC. Sequence and numbering of the oligonucleotide used for NMR studies is reported in Fig. 4e, while folding topology of the major conformation of *c-MYC* determined by NMR by Ambrus *et al.* is shown in Fig. 4d)^13^. The proton resonance assignment of the *c-MYC* DNA in 90% H_2_O/10% D_2_O was performed on the basis of the assignment reported in literature.^13^NMR samples were referenced with 2,2-dimethyl-2-silapentane-5-sulphonate (DSS) and prepared in buffer containing 25 mM tris·HCl at pH 7.4 with or without additional 100 mM KCl, in 90%H_2_O/10% D_2_O. ^1^H NMR spectra were recorded on DNA and DNA-ligand complexes with gradient-assisted excitation sculpting^45^ or jump-return-Echo^46^ for water suppression.

#### Cell viability analysis using MTT assay

The human hepatocellular carcinoma cells (HepG2), human breast cancer cells (MCF 7), human colon carcinoma cells (HCT 116) and mouse normal myoblast cells (C2C12) were cultured in DMEM containing high glucose (5.5 mM) supplemented with 10% FBS at pH 7.4. Cells were maintained in tissue culture plates containing 4 × 10^5^ cells/well at 37 °C in an atmosphere of 5% carbon dioxide (CO_2_)/95% air. The MTT cell proliferation assay determines the ability of living cells to reduce the yellow tetrazole, 3-(4,5-dimethylthiazol-2-yl)-2,5-diphenyltetrazolium bromide (MTT) to purple formazan crystals by mitochondrial enzymes. For the MTT assay, HepG2 cancer cells were treated with various concentrations of carbazole derivatives **5** and **BTC a**–**g** for 24 h. Following incubation with each compound for 24 h, 20 *μ*L of MTT was added (at a concentration of solution 5 mg/mL in phosphate-buffered saline, pH 7.4.) to each well. After incubation for 4 h at 37 °C, the culture medium was removed, and the formazan crystals were dissolved in 200 μL DMSO. Absorbance (A) of formazan dye was measured at 570 nm using a micro plate reader. The background absorbance was determined at 690 nm and subtracted from the 570 nm measurement. The percentage of viable cells was determined by the equation (2):
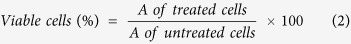
Following the similar protocol, MCF 7, HCT 116 and C2C12 were treated with various concentrations of **BTC f** and the percentage of viable cells was determined.

#### qRT-PCR analysis

To evaluate the role of **BTC** at the transcriptional level of *c-MYC*, qRT-PCR was performed. Cancer cells were incubated with various concentrations (1.0, 2.5 and 5.0 μM) of **BTC c** and **BTC f** for 24 h at 37 °C in humidified 5% CO_2_ incubator. Total RNA was prepared from compound treated and untreated HepG2 cells using the Trizol kit according to the manufacturer’s protocol (Invitrogen Corporation). cDNA library was prepared by High Capacity cDNA Reverse Transcription Kit (Applied Biosystems). Master mix (2X) was prepared by 2 *μ*L 10X RT buffer, 0.8 *μ*L 25 × 100 mM dNTP mix, 2 *μ*L 10X RT random primer, 1 *μ*Lof reverse transcriptase, 1 *μ*L of RNase inhibitor and 3.2 *μ*L nuclease free water. 10 *μ*L 2X master mix was added to 10 *μ*L sample and sealed in 96 well plates. Reverse transcriptase reaction was performed on a Light Cycler 480 II (Roche). The thermal cycling condition was programmed as 10 min at 25 °C, 120 min at 37 °C and 5 min at 85 °C for one single cycle. qRT-PCR was performed on a Light Cycler 480 II (Roche) with SYBR green JumpStart Taq ReadyMix (Sigma, Saint Louris, USA) reagent using the cDNA library as template. The primers used for the qRT-PCR analyses had the following sequences^47^,^48^:

*c-MYC* (forward): 5′-CTGCGACGAGGAGGAGGACT-3′

*c-MYC* (reverse): 5′- GGCAGCAGCTCGAATTTCTT-3′

GAPDH (forward): 5′-GACGGCCGCATCTTCTTGT-3′

GAPDH (reverse): 5′-CACACCGACCTTCACCATTTT-3′

The PCR mixture (25 *μ*L) contained 15 pmol of each primer, 7 *μ*L of water, 5 μL of cDNA, 12.5 *μ*L 2X JumpStart Taq ReadyMix. The samples were placed in 96-well plates (Roche), and PCR amplification was performed using Light Cycler 480 II real-time PCR detection system (Roche). The thermal cycling conditions were 2 min at 94 °C and then 40 cycles of 15 s at 94 °C, followed 60 s at 60 °C. We used the comparative cycle threshold method (*C*_T_ method) for relative quantification of gene expression^49^. The *C*_T_ for the target and the *C*_T_ for the internal control (GAPDH) were determined for **BTC c** or **BTC f** or untreated (control) samples. Finally, the arithmetic calibrator (2^−ΔΔ*C*T^) was used to calculate the relative mRNA level expression of *c-MYC*. Difference in *c-MYC* expression was expressed as fold changes.

#### Western blot analysis

Hepatocellular carcinoma HepG2 cells were treated with different concentration (1.0, 2.5 and 5.0 μM) of **BTC c** and **BTC f** for 24 h at 37 °C in humidified CO_2_ incubator. After the incubation period, cells were washed once with PBS (pH 7.4) and lysed with cold cell lysis buffer (20 mM Tris, 100 mM NaCl, 1 mM EDTA in 0.5% Triton X-100). Cell lysate were collected from the treated and untreated cells, and the total protein content was estimated by Lowry method^50^. Equal amount of proteins (60 *μ*g) from the cell lysate were separated by 10% SDS–PAGE and transferred to nitrocellulose membranes. The membranes were blocked, washed and probed using antibodies directed against *c-MYC* and GAPDH (as loading control) for overnight at room temperature. The blots were washed and immunoreactive bands were incubated with a 1:2000 dilution of ALKP conjugated secondary antibody for 2 h at room temperature. Binding signals were visualized with NBD/BCIP substrate. Relative band intensities were determined by using ImageJ software.

#### Flow cytometric determination of the cell cycle histogram by PI staining

Cell cycle histogram was analysed using propidium iodide (PI) staining by Flowcytometry. HepG2 cells (1 × 10^6^) per 60 mm petridish (~80% confluence) were treated with various concentration of **BTC f** (1.0, 2.5, 5.0 μM) for 24 h in fresh growth medium. Cells were harvested by trypsinization, resuspended in PBS and fixed with 2 mL of ice-cold 70% ethanol for overnight at 4 °C. The pellets were collected by centrifugation and resuspended in PBS solution, containing 10 *μ*g/mL PI (Sigma) and 10 *μ*g/mL RNaseA (Sigma). After incubation for 30 minutes in the dark at 37 °C, cells were analyzed for DNA content using a FACS flow cytometer (BD Biosciences). Cell distribution among cell cycle phases and the percentage of apoptotic cells were evaluated using Cell-Quest Pro software (BD).

#### Flowcytometric assay of apoptosis

Annexin V–FITC and propidium iodide (PI) stains were used to determine the percentage of cells undergoing apoptosis and necrosis. An apoptosis assay was conducted using the protocol supplied by the manufacturer. Briefly, 1 × 10^6^ HepG2 cells per 60 mm petridish (~90% confluence) were treated with different concentration of **BTC f** (1.0, 2.5, 5.0 μM) for 24 h in fresh growth medium. Cells were then harvested with trypsinization. After centrifugation at 700 rpm for 5 min at 4 °C, cell pellet was suspended in 500 μL 1 × binding buffer and then treated with 5 μL Annexin V–FITC and 5 μL PI. After incubation for 5 min on ice, each sample was analysed immediately using fluorescence–activated cell sorting (FACS) analysis (BD Biosciences, Mountain View, CA, USA). Approximately 10,000 HepG2 cells were detected for each sample. Cytogram analysis was done using the Cell Quest software.

### Confocal microscopy

Cellular localization of **BTC f** was monitored by live cell imaging. HepG2 cells (1 × 10^3^) were cultured on 35 mm diameter glass-bottomed cover slips for 24 h followed by incubation with **BTC f** (5.0 μM) for 4 h inside CO_2_ (5%) incubator at 37 °C. After incubation, cells were washed with PBS three times to remove the excess ligand and bathed in DMEM (2 mL) before imaging. Localization of **BTC f** was viewed under confocal microscope (Zeiss LSM 510 laser scanning microscope, Standort Göttingen, Germany). At least 5 fields per slide and three independent sets were examined.

### Statistical analysis

All data were given as mean ± S.D. Differences between two groups were compared by unpaired Student’s t-test. For multi-group comparisons, analysis of variance was determined by ANOVA. A value of P < 0.05 was considered statistically significant. The statistical analysis was done by using GraphPad Instant Software (Graph-Pad, La Jolla, CA, USA).

## Additional Information

**How to cite this article**: Panda, D. *et al.* A Nucleus-Imaging Probe That Selectively Stabilizes a Minor Conformation of *c-MYC* G-quadruplex and Down-regulates *c-MYC* Transcription in Human Cancer Cells. *Sci. Rep.* **5**, 13183; doi: 10.1038/srep13183 (2015).

## Supplementary Material

Supplementary Information
