# Supplementary material for: A Nucleus-Imaging Probe That Selectively Stabilizes a Minor Conformation of c-MYC G-quadruplex and Down-regulates c-MYC Transcription in Human Cancer Cells
Source: Sci Rep. 2015 Aug 19;5:13183. doi: 10.1038/srep13183 (PMC4541407; doi:10.1038/srep13183)
Supplement: Supplementary Information [file srep13183-s1.doc]

**SUPPLEMENTARY INFORMATION**

A Nucleus-Imaging Probe That Selectively Stabilizes a Minor Conformation of *c-MYC* G-quadruplex and Down-regulates *c-MYC* Transcription in Human Cancer Cells

Deepanjan Panda,^1^ Manish Debnath,^1^ Samir Mandal,^1^ Irene Bessi,^2^ Harald Schwalbe,^2^ Jyotirmayee Dash^1^*

^1^Department of Organic Chemistry, Indian Association for the Cultivation of Science, Jadavpur, Kolkata-700032, India; email: [ocjd@iacs.res.in](mailto:ocjd@iacs.res.in)

^2^Institute of Organic Chemistry and Chemical Biology, Goethe University Frankfurt and Centre for Biomolecular, Magnetic Resonance, Max-von-Laue Strasse 7, 60438, Frankfurt am Main, Germany

**Contents**

1.0 General Information S3

2.0 Synthesis of azide building blocks S4

3.0 FRET melting analysis S7

4.0 Fluorimetric titration S8

5.0 CD spectroscopy S10

6.0 UV-Vis spectroscopy S11

7.0 NMR spectroscopy S12

8.0 MTT assay for cytotoxicity S13

9.0 NMR spectra of carbazole derivatives S14

10.0 References S25

**1.0 General Information**

All solvents and reagents were purified by standard techniques reported in Armarego, W. L. F., Chai, C. L. L., Purification of Laboratory Chemicals, 5th edition, Elsevier, 2003; or used as supplied from commercial sources (Sigma-Aldrich Corporation® unless stated otherwise). All reactions were generally carried out under inert atmosphere unless otherwise noted.TLC was performed on Merck Kieselgel 60 F254 plates, and spots were visualized under UV light.Products were purified by flash chromatography on silica gel (100-200 mesh, Merck). ^1^H and ^13^C NMR spectra were recorded on either Brüker ADVANCE 500 (500MHz and 125 MHz), or JEOL 400 (400 MHz and 100 MHz) instruments using deuterated solvents as detailed and at ambient probe temperature (300 K). Chemical shifts are reported in parts per million (ppm) and are referred to the residual solvent peak. The followingnotations are used: singlet (s); doublet (d); triplet (t); quartet (q); multiplet (m); broad(br). Coupling constants are quoted in Hertz and are denoted as *J*. Mass spectra wererecorded on a Micromass® Q-Tof (ESI) spectrometer. All DNA oligonucleotides were purchased from Sigma-Aldrich.

**2.0 Synthesis of azide building blocks**

**Phenyl azide (6a):** Aniline (3.0 g, 32.21 mmol) was suspended in 17% hydrochloric acid (25 mL) at room temperature. The solution was cooled to 0 °C using an ice bath. Then NaNO_2_ (3.33 g, 48.32 mmol) in water (10 mL) was added in small portions. After stirring at 0 °C for 20-30 min, an aqueous solution of NaN_3_ (3.14 g, 48.32 mmol) was slowly added and the mixture was stirred for additional 2 h at room temperature. After completion, the reaction mixture was extracted with diethyl ether (3 x 40 mL). The combined organic fractions were washed with saturated NaHCO_3_ solution (2 x 40 mL) and brine (2 x 40 mL). The organic fractions were dried over Na_2_SO_4_ and evaporated under reduced pressure to give the azide **6a** (2.69 g,70%) as a colorless oil^1^. ^1^H NMR (400 MHz, CDCl_3_): 7.36 (t, 2H, *J* = 7.6 Hz), 7.15 (td, 1H, *J* = 7.6, 0.7 Hz), 7.04 (dd, 2H, *J* = 7.6, 0.7 Hz); ^13^C NMR (100 MHz,CDCl_3_): 139.9, 129.7, 124.8, 119.0.

**4-Azidophenol (6b):** 4-Aminophenol (3.51 g, 32.21 mmol) was suspended in 17% hydrochloric acid (25 mL) at room temperature. The solution was cooled to 0 °C using an ice bath. Then NaNO_2_ (3.33 g, 48.32 mmol) in 10 mL water was added in small portions. After stirring at 0 °C for 20-30 min, an aqueous solution of NaN_3_ (3.14 g, 48.32 mmol) was slowly added and the mixture was stirred for additional 2 h at room temperature. After completion, the reaction mixture was extracted with diethyl ether (3 x 10 mL). The combined organic fractions were washed with saturated NaHCO_3_ solution (2 x 10 mL) and brine (2 x 40 mL). The organic fractions were dried over Na_2_SO_4_ and evaporated under reduced pressure to give the azide **6b** (3.7g, 85%). Spectroscopic data were in agreement with previous reports^2^.

**4-Azidoaniline (6c):** A mixture of 4-bromo aniline (500 mg, 2.9 mmol), NaN_3_ (5.8 mmol), sodium ascorbate (0.1 mmol), CuI (0.2 mmol), DMEDA (0.3 mmol) in 5 mL EtOH–H_2_O (7:3) were heated at 80 °C until the completion of the starting bromide (monitored by TLC). The reaction mixture was cooled to room temperature, extracted with diethyl ether and purified by flash chromatography to give the azide **6c** (291 mg, 75%) as a brown solid^3^. ^1^H NMR (400 MHz, CDCl_3_): 6.84 (d, 2H, *J* = 10.7 Hz), 6.67 (d, 2H, *J* = 10.9 Hz), 3.65 (s_br_, 2H); ^13^C NMR (100 MHz, CDCl_3_): 143.7, 130.0, 119.9, 116.2.

**4-Azidobenzaldehyde (6d):** A mixture of 4-bromo benzaldehyde (1.0 g, 5.4 mmol), NaN_3_ (10.8 mmol), sodium ascorbate (0.1 mmol), CuI (0.2 mmol), DMEDA (0.3 mmol) in 5 mL EtOH–H_2_O (7:3) were heated at 80 °C until the completion of the starting bromide (monitored by TLC). The reaction mixture was cooled to room temperature, extracted with diethyl ether and purified by flash chromatography to give the azide **6d** as a yellow oil; 37% purity by ^1^H NMR; 30% overall yield. Spectroscopic data were in agreement with previous reports^4^.

**4-Azidobenzoic acid (6e):** 4-Amino benzoic acid (1.0 g, 7.29 mmol) was suspended in 17% hydrochloric acid (25 mL) at room temperature. The solution was cooled to 0 °C using an ice bath. Then NaNO_2_ (754.5 mg, 10.94 mmol) in 5 mL water was added in small portions. After stirring at 0 °C for 20-30 min, an aqueous solution of NaN_3_ (710.78 mg, 10.94 mmol) was slowly added and the mixture was stirred for additional 2 h at room temperature. After completion, the reaction mixture was extracted with diethyl ether (3 x 10 mL). The combined organic fractions were washed with saturated NaHCO_3_ solution (2 x 40 mL) and brine (2 x 40 mL). The organic fractions were dried over Na_2_SO_4_ and evaporated under reduced pressure to give the azide **6e** (927 mg, 78%) as a light brown solid^5^. ^1^H NMR (400 MHz DMSO-d_6_): 12.98 (s, 1H), 7.97–7.94 (m, 2H), 7.23–7.20 (m, 2H); ^13^C NMR: (125 MHz, DMSO-d_6_): 166.5, 143.9, 131.2, 127.3, 119.1.

**4-Azido-N-(3-(dimethylamino)propyl)benzamide (6f)**. A mixture of 4-azido benzoic acid **6e** (500 mg, 3.1 mmol) and HOBt (612.53 mg, 4.0mmol) was dissolved in 10 mL CH_2_Cl_2_. After cooling to 0 °C, DCC (825.32 mg, 4.0 mmol) and 3-(dimethylamino)-propylamine (0.513 mL, 2.8 mmol) were added, and the mixture was stirred overnight at room temperature. CH_2_Cl_2_ was evaporated in vacuo. The crude product was purified by column chromatography (CH_2_Cl_2_:100% then CH_2_Cl_2_/MeOH (20:1) to give the azide **6f** (655 mg, 86%) as a colorless liquid. ^1^H NMR (400 MHz, DMSO-d_6_): 8.60 (t, 1H, *J* = 6.9 Hz), 7.90 (d, 2H, *J* = 11.3 Hz), 7.17 (d, 2H, *J* = 11.7 Hz), 3.26 (t, 2H, *J* = 7.8 Hz), 2.39 (t, 2H, *J* = 9.8 Hz), 2.23 (s, 6H), 1.69 (t, 2H, *J* = 9.3 Hz); ^13^C NMR (125 MHz, DMSO-d_6_): 165.1, 142.1, 131.1, 129.0, 118.8, 56.5, 44.5, 37.5, 26.5; HRMS (ESI) calculated for [C_12_H_18_N_5_O]: 248.1506, Found 248.1508.

**2-Azido ethanol (6g):** Chloroethanol (165 mg, 2.05 mmol) was added rapidly to a solution of sodium azide (157 mg, 2.42 mmol) in 5 mL water at room temperature. The reaction mixture was stirred at 30 °C for 1 h and then at 70 °C for 72 h. The resulting solution was cooled to room temperature, saturated with sodium sulfate, and extracted with dichloromethane (10 mL). The combined organic layers were dried over anhydrous sodium sulfate and concentrated on a rotary evaporator to give crude 2-azidoethanol **6g** (166 mg, 93%) as a colorless liquid. Spectroscopic data were in agreement with previous reports^6^.


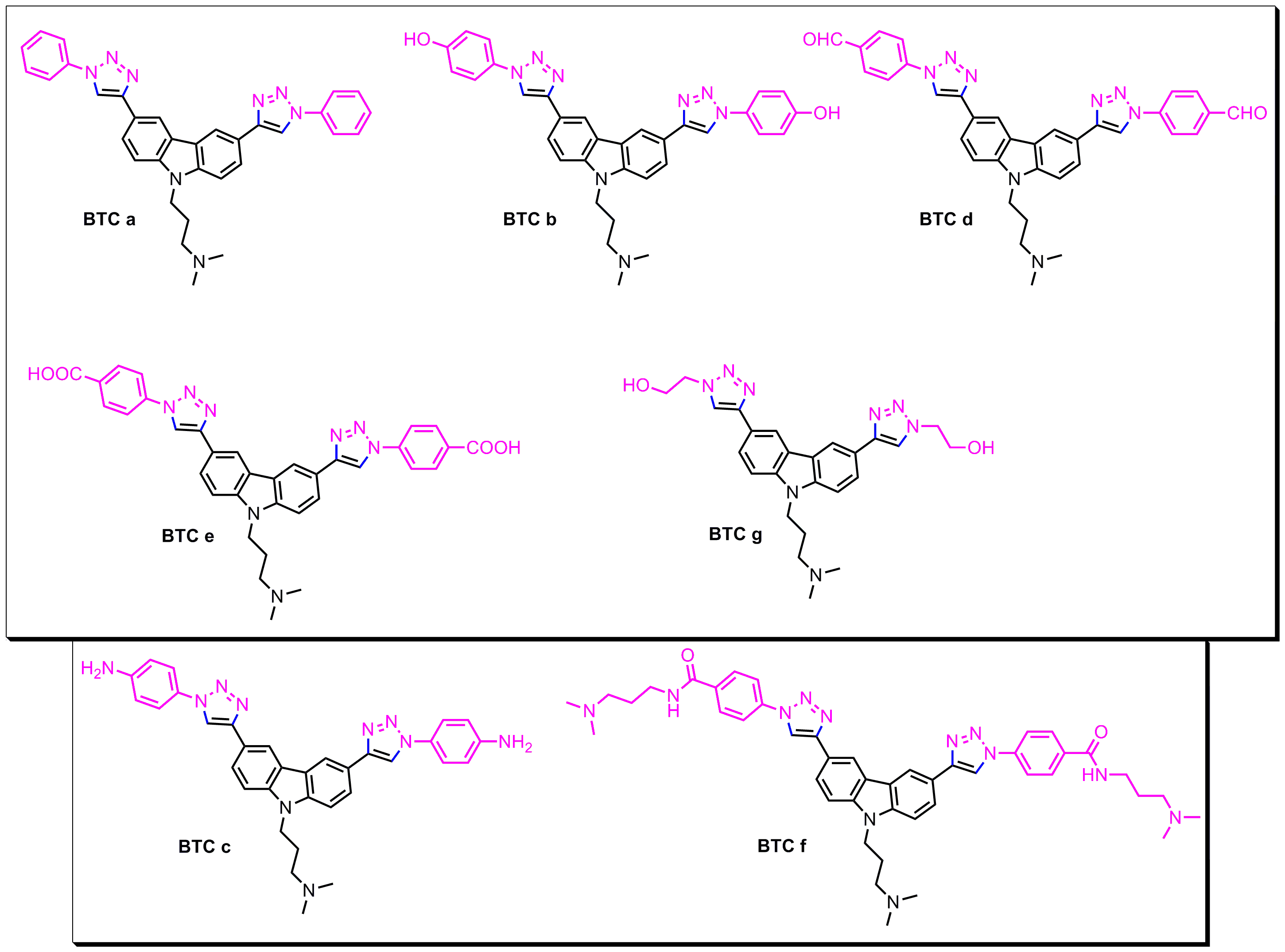


**Chart S1. Bis-triazolyl carbazole derivatives.**

**3.0 FRET melting analysis**

**Table S1. FRET stabilization potential for carbazole derivatives 5 and BTC a-g in 50 mM potassium cacodylate buffer, pH 7.4.**

| **Ligands (1 *µ*M)** | **ΔTm/K of Ligands** | | |
| --- | --- | --- | --- |
|  | *c-MYC* | *c-KIT 1* | *c-KIT 2* |
| **5** | 0.3 ± 0.1 | 0.7 ± 0.2 | 0.4 ± 0.1 |
| **BTC a** | 8.1 ± 0.9 | 26.6 ± 1.9 | 22.7 ± 2.1 |
| **BTC b** | 3.5 ± 0.7 | 17 ± 1.4 | 19 ± 1.5 |
| **BTC c** | 18.9 ± 1.6 | 30.2 ± 1.8 | 22.6 ± 1.6 |
| **BTC d** | 10.6 ± 1.1 | 12.2 ± 1.2 | 10 ± 1.1 |
| **BTC e** | 0.6 ± 0.1 | 1.4 ± 0.1 | 1.8 ± 0.1 |
| **BTC f** | 22.7 ± 1.4 | 39.4 ± 2.5 | 23.2 ± 1.6 |
| **BTC g** | 1.9 ± 0.2 | 12.4 ± 0.5 | 0.8 ± 0.1 |

**
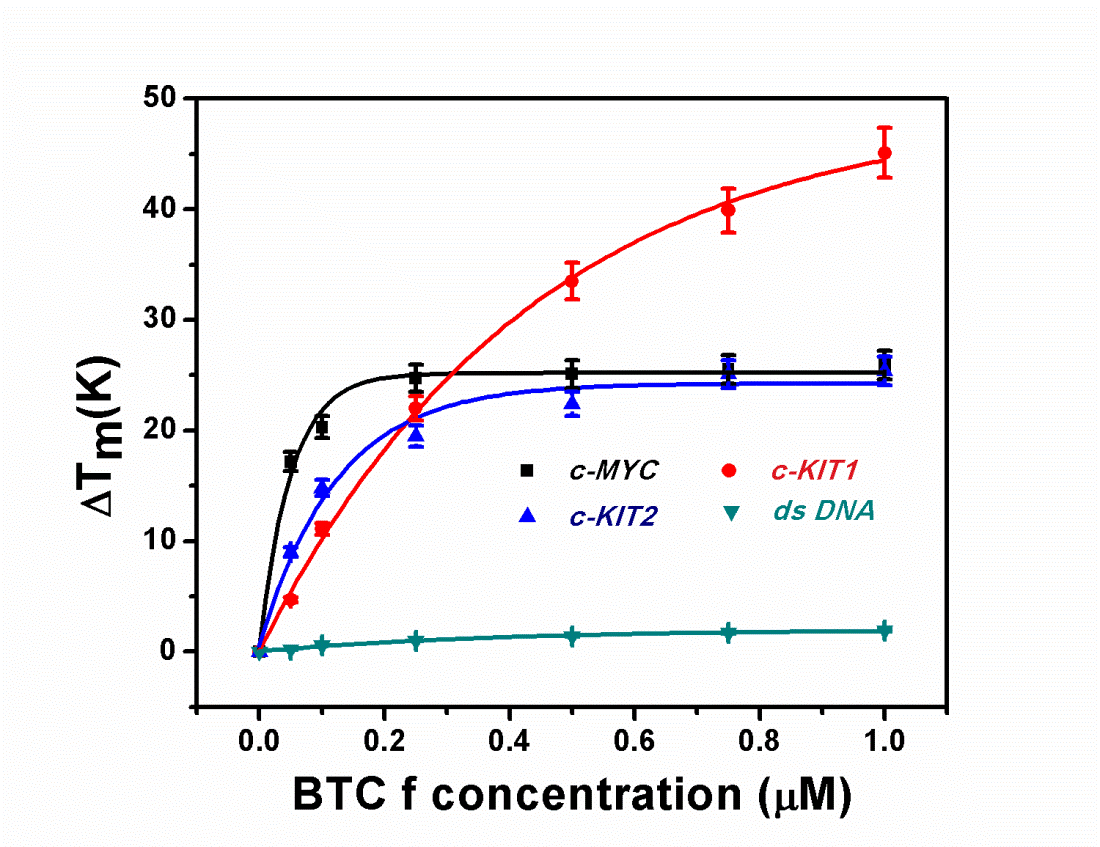
**

**Figure S1.** FRET stabilization curves of **BTC f** upon binding to *c-MYC* = (69.7 ± 1.4), *c-KIT1* = (52.01 ± 1.9), *c-KIT2* = (68.4 ± 1.6) and *ds* DNA = (60.3 ± 1.9) in MQ water, pH 7.4. Saturation concentration of **BTC f** (*µ*M) for stabilization of G-quadruplexes (200 nM) was determined in the absence of K^+^.

**Table S2.** Saturation concentration of **BTC f** (*µ*M) required to achieve maximum stabilization as determined by FRET melting.

|  | Saturation concentration of **BTC f** (nM) | | | |
| --- | --- | --- | --- | --- |
|  | *c-MYC* | *c-KIT1* | *c-KIT2* | *ds DNA* |
| In the presence of 50 mM K^+^ | 100 | 750 | 500 | NA |
| In the absence of K^+^ | 250 | 1000 | 750 | NA |

**4.0 Fluorimetric titration**


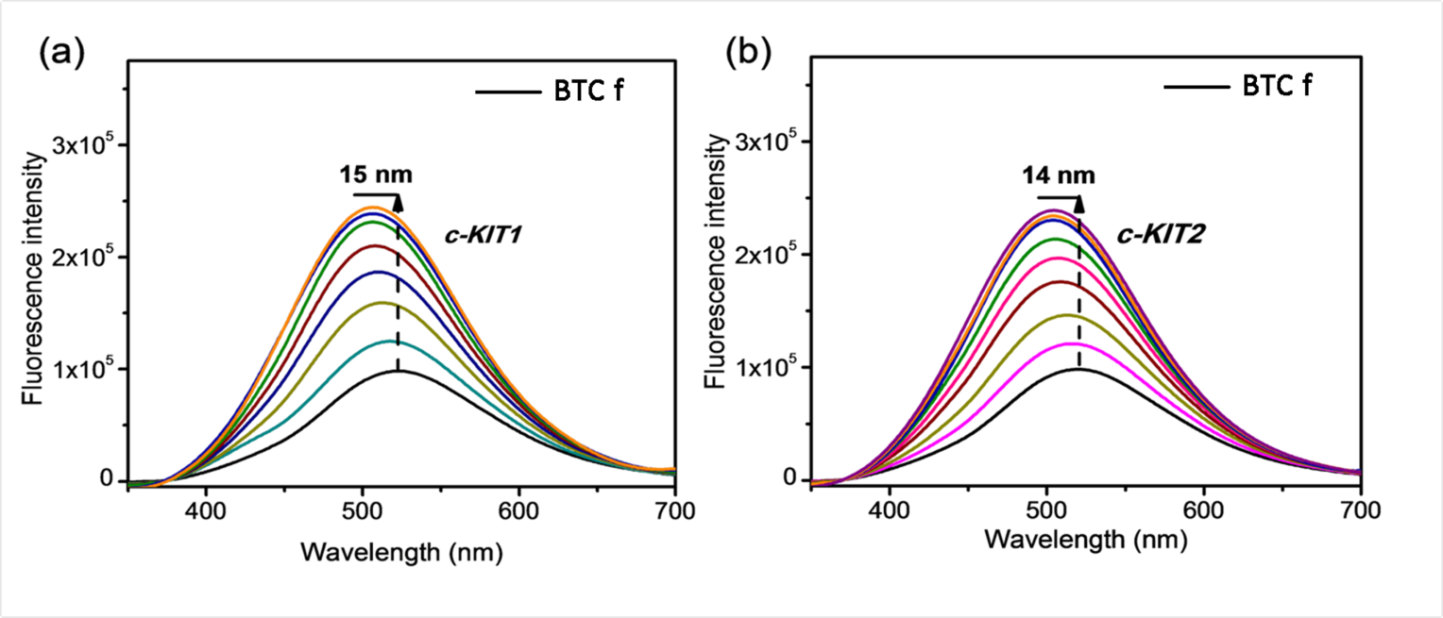


**Figure S2.** Fluorimetric titration spectra of **BTC f** (1.0 *µ*M) with (0-6 eq): (a) *c-KIT1* and (b) *c-KIT2*, in 100 mM Tris•HCl buffer containing 100 mM KCl at pH 7.4.

**Table S3.** Binding constants (*K*_d_) obtained by fluorescence titration studies.

| **Sequences** | ***K_d_* (*µ*M)** | **F/F_0_** | **Blue shift (nm)** |
| --- | --- | --- | --- |
| *c-MYC* | 0.3 ± 0.015 | 3.25 | 20 |
| *c-MYC unfolded* | n.d. | 5.35 | 25 |
| *c-KIT 1* | 1.38 ± 0.07 | 2.5 | 15 |
| *c-KIT 2* | 1.37 ± 0.06 | 2.45 | 14 |
| *ds DNA* | > 10.0 | 1.27 | 5 |


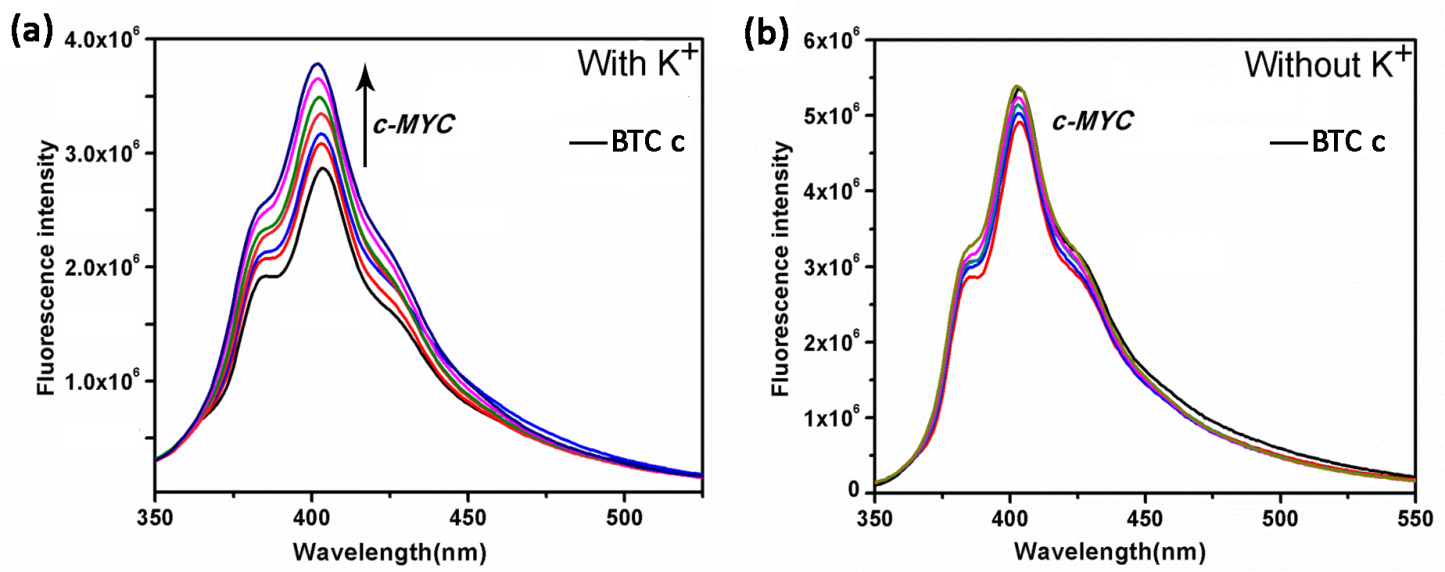


**Figure S3.** Fluorimetric titration spectra of **BTC c** (1.0 *µ*M) with (0-6 eq): (a) *c-MYC* in 100 mM Tris•HCl buffer at pH 7.4 containing 100 mM KCl and (b) *c-MYC* in 100 mM Tris•HCl buffer at pH 7.4 without KCl.


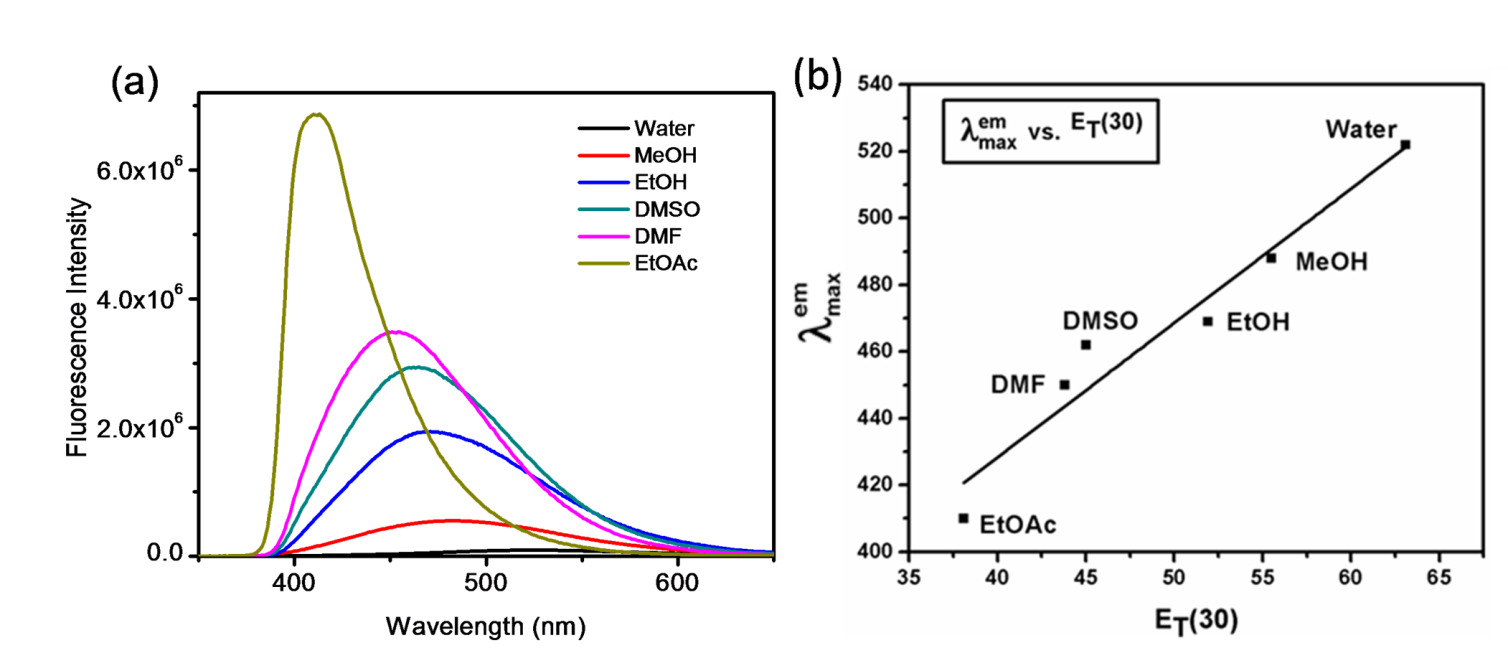


**Figure S4.** (a) Environment polarity effect of **BTC f** in various solvent environments (ethyl acetate, DMF, DMSO, ethanol, methanol, water). (b) Plot of emission maxima (
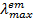
) of **BTC f** in different solvents *vs.* E_T_(30) of different solvents.

**Table S4.** Excitation (
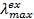
) and emission (
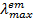
) maximum wavelengths of **BTC f** and Stokes Shift values (S) in solvent environments.

| **Environment** | 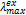**(nm)** | 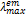**(nm)** | **S (cm^−1^)** |
| --- | --- | --- | --- |
| Ethyl acetate | 290 | 411 | 10152 |
| DMF | 290 | 452 | 12359 |
| DMSO | 290 | 461 | 12791 |
| Ethanol | 290 | 470 | 13206 |
| Methanol | 290 | 488 | 13991 |
| Water | 290 | 522 | 15326 |

**5.0 CD spectroscopy**


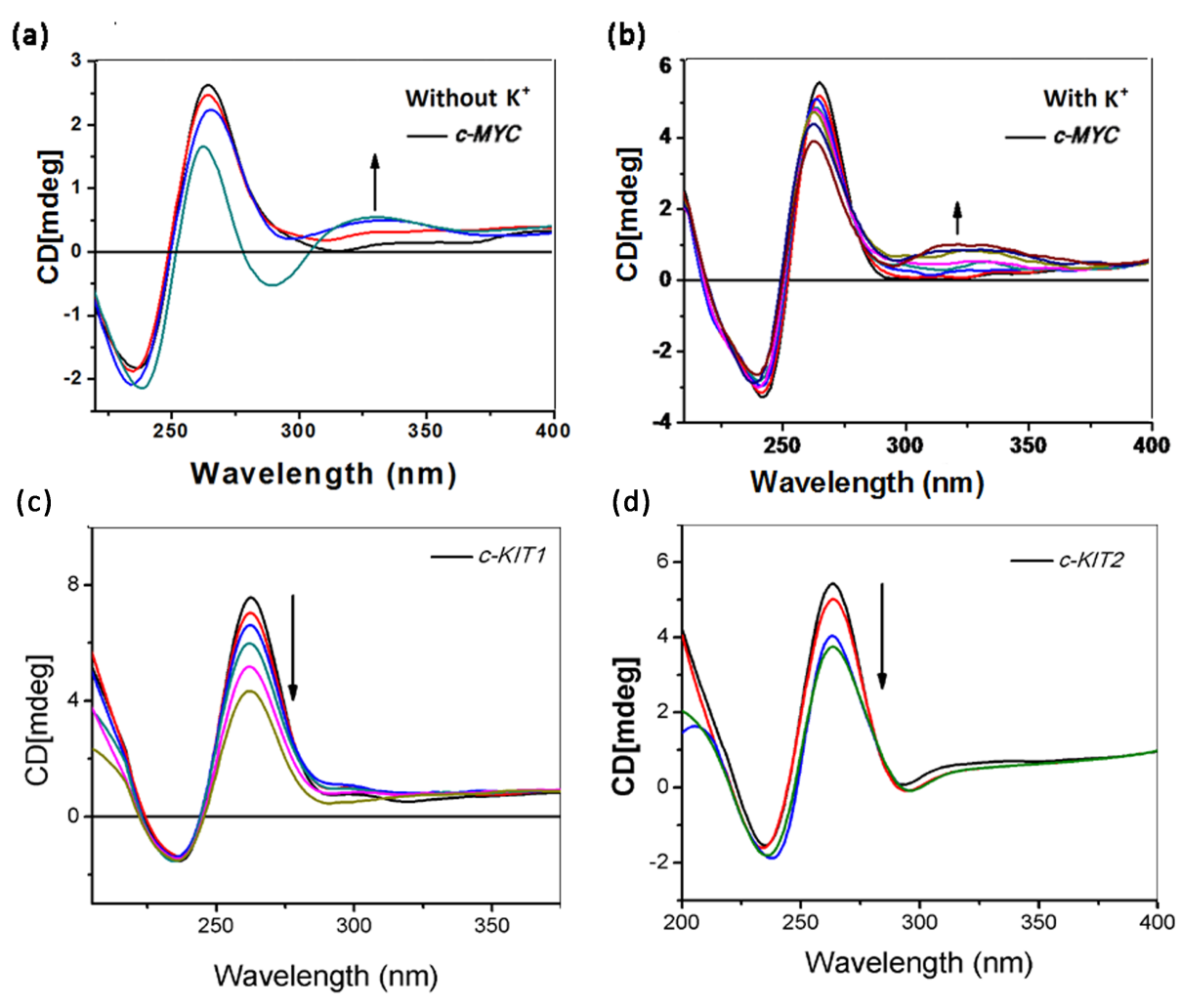


**Figure S5.** CD spectra of: (a) *c-MYC* (10 *μ*M) in 100 mM Tris•HCl buffer (pH 7.4) without KCl, (b) *c-MYC* (10 *μ*M) in 100 mM Tris•HCl buffer (pH 7.4) containing 100 mM KCl, (c) *c-KIT1* (10 *μ*M) in 100 mM Tris•HCl buffer (pH 7.4) containing 100 mM KCl and (d) *c-KIT2* (10 *μ*M) in 100 mM Tris•HCl buffer (pH 7.4) containing 100 mM KCl; each titrated with 0-3 eq of ligand **BTC f**.

**6.0 UV-Vis spectroscopy**

**
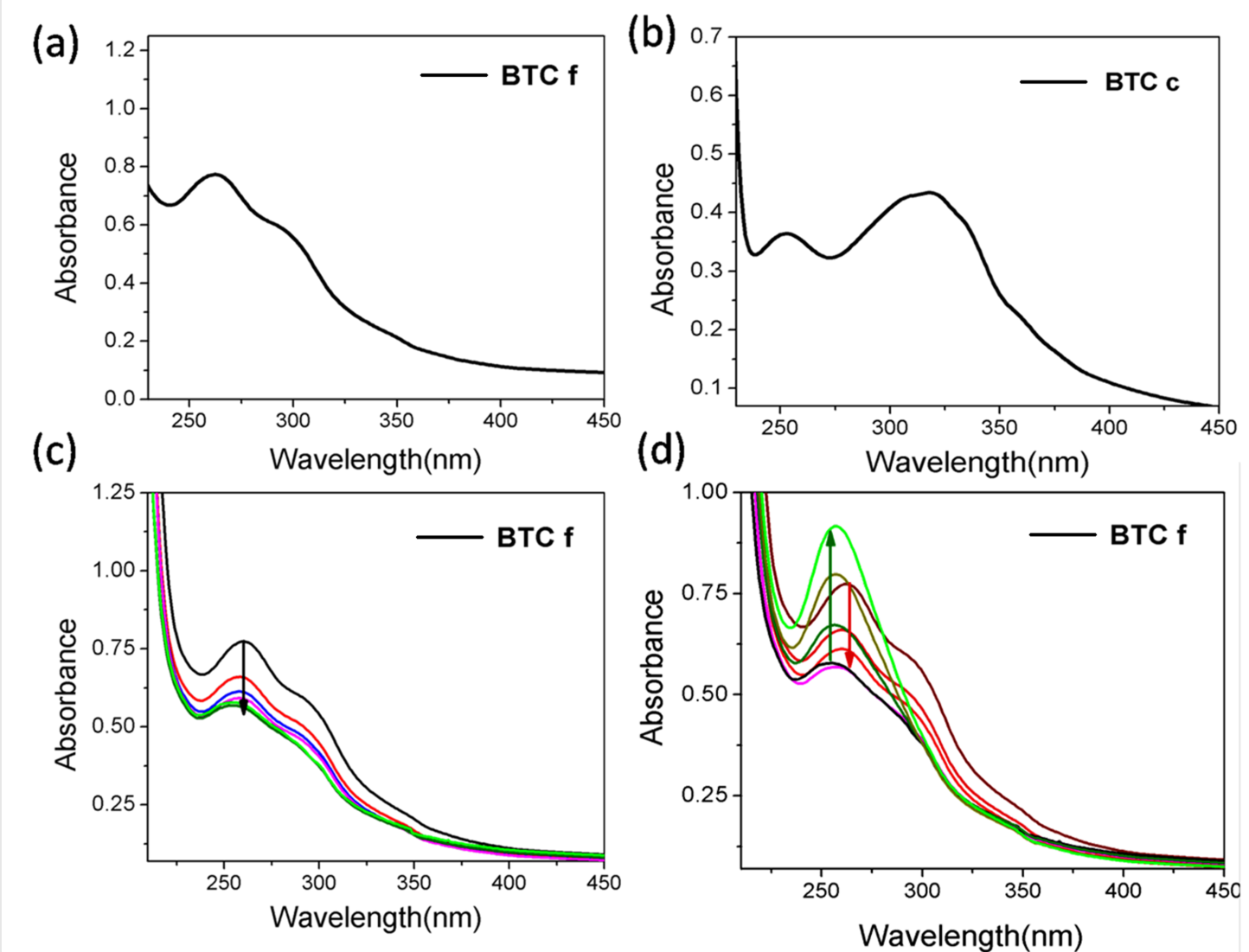
**

**Figure S6.** UV-Vis spectra of (a) **BTC f** (25 µM), (b) **BTC c** (25 µM), (c) hypochromicity changes observed on step-wise addition of *c-MYC* (0-0.4 equiv.) to **BTC f** (25 µM) and (d) step-wise addition of *c-MYC* (0-1.0 equiv.) showing overlap of *c-MYC* DNA in 100 mM Tris-KCl buffer containing 100 mM KCl, pH 7.4.

**7.0 NMR spectroscopy**


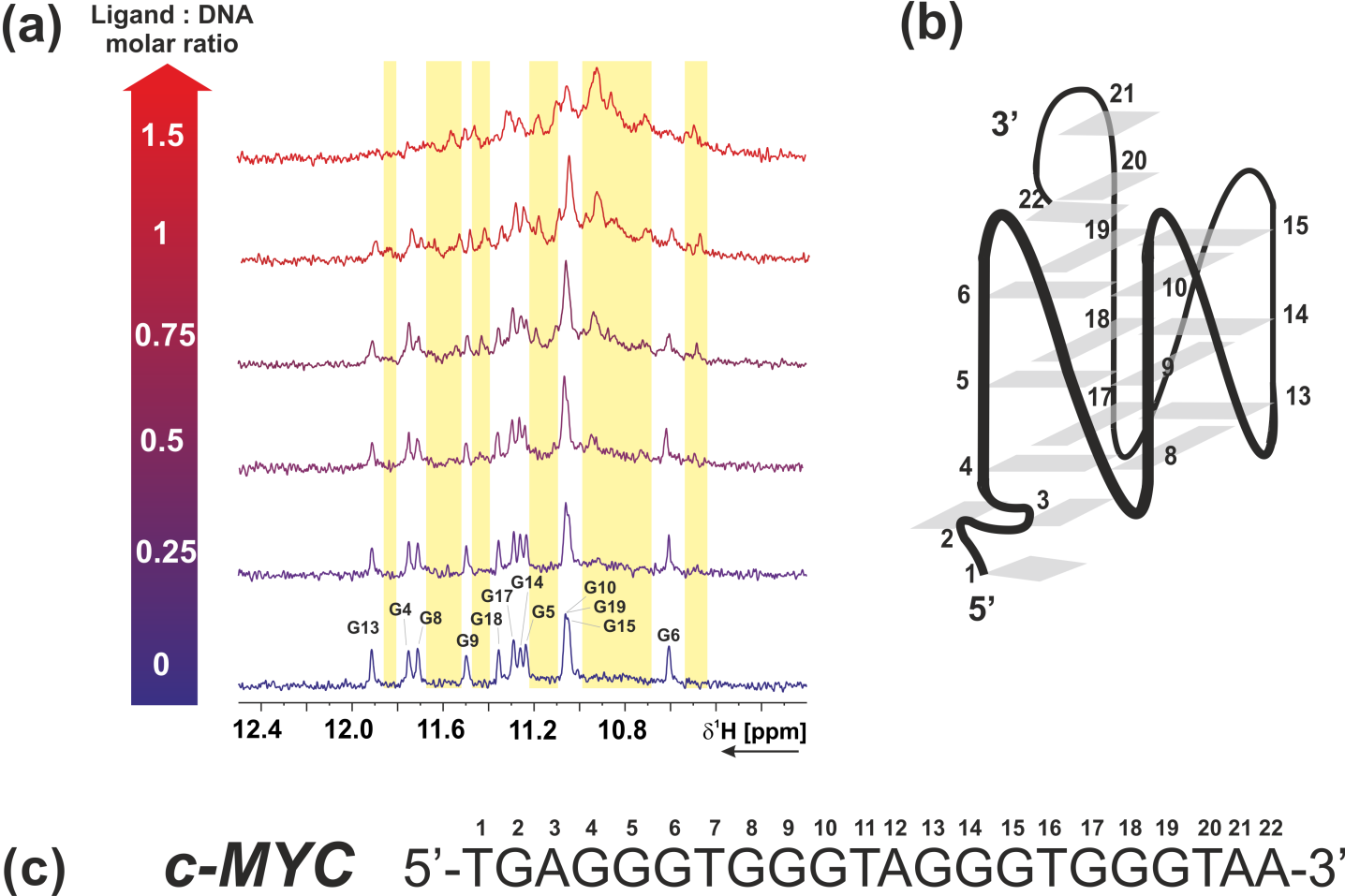


**Figure S7**. Imino region of 100 *μ*M *c-MYC* in the presence of **BTC c** at different [ligand]:[DNA] molar ratio, in 25 mM Tris⋅HCl buffer at pH 7.4 containing 100 mM KCl, 90%H_2_O/10% D_2_O. Titrations were performed at 298 K, 600 MHz. Signals from the major conformation of *c‑MYC* are labeled according to numbering reported in panel (c) and signals from the minor conformation(s) stabilized by **BTC c** are highlighted in yellow. (b) Scheme of the major conformation (non binding‑competent) of *c‑MYC* determined by NMR from Ambrus *et al*. (c) *c-MYC* sequence used for NMR binding titration.

**8.0 MTT assay for cytotoxicity**

**Table S5.** IC_50_ values of carbazole derivatives after treatment in hepatocellular carcinoma cells (HepG2).

|  | **IC_50_ of carbazolederivatives (*µ*M)** | | | | | | | |
| --- | --- | --- | --- | --- | --- | --- | --- | --- |
|  | **5** | **BTC a** | **BTC b** | **BTC c** | **BTC d** | **BTC e** | **BTC f** | **BTC-g** |
| HepG2 | > 100 | 26.6 ± 3.6 | 38.9 ± 5.8 | 11.4 ± 1.5 | 69.6 ± 8.5 | > 100 | 4.3 ± 0.69 | > 100 |

**
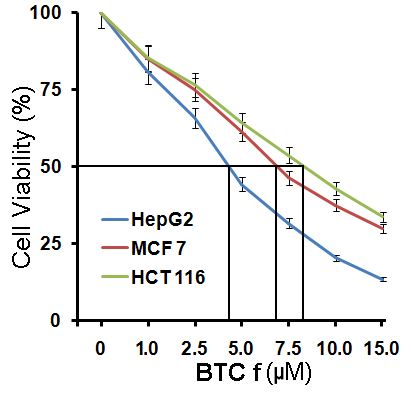
**

**Figure S8.** Dose response curves for MTT toxicity assay to determine IC_50_ values upon treatment of **BTC f** to HepG2, MCF 7 and HCT 116 cells. Cells (1×10^4^ HepG2 cell/ well) were treated with different dose (0-15 *μ*M) of **BTC f** for 24 h.

**9.0 NMR spectra of carbazole derivatives**

**^1^H and ^13^C NMR of 1:**

**
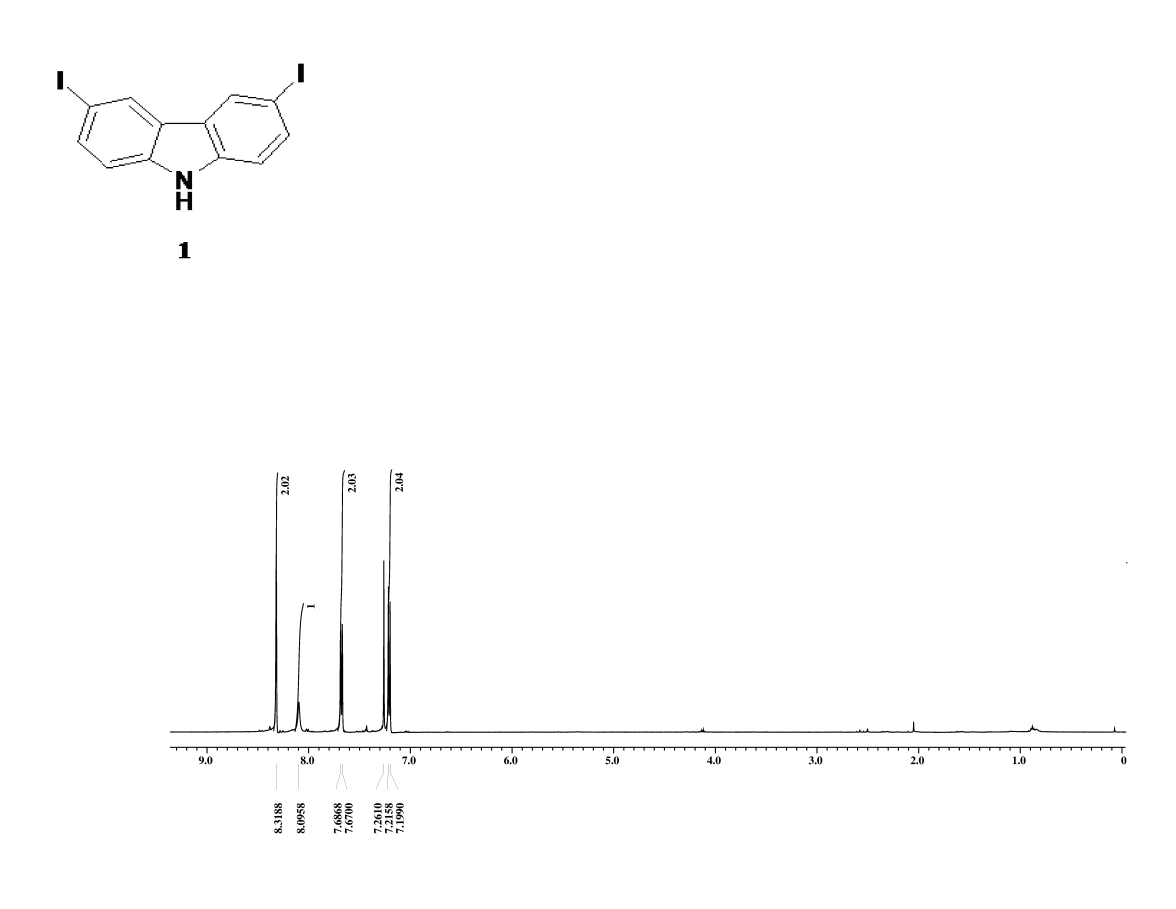
**

**
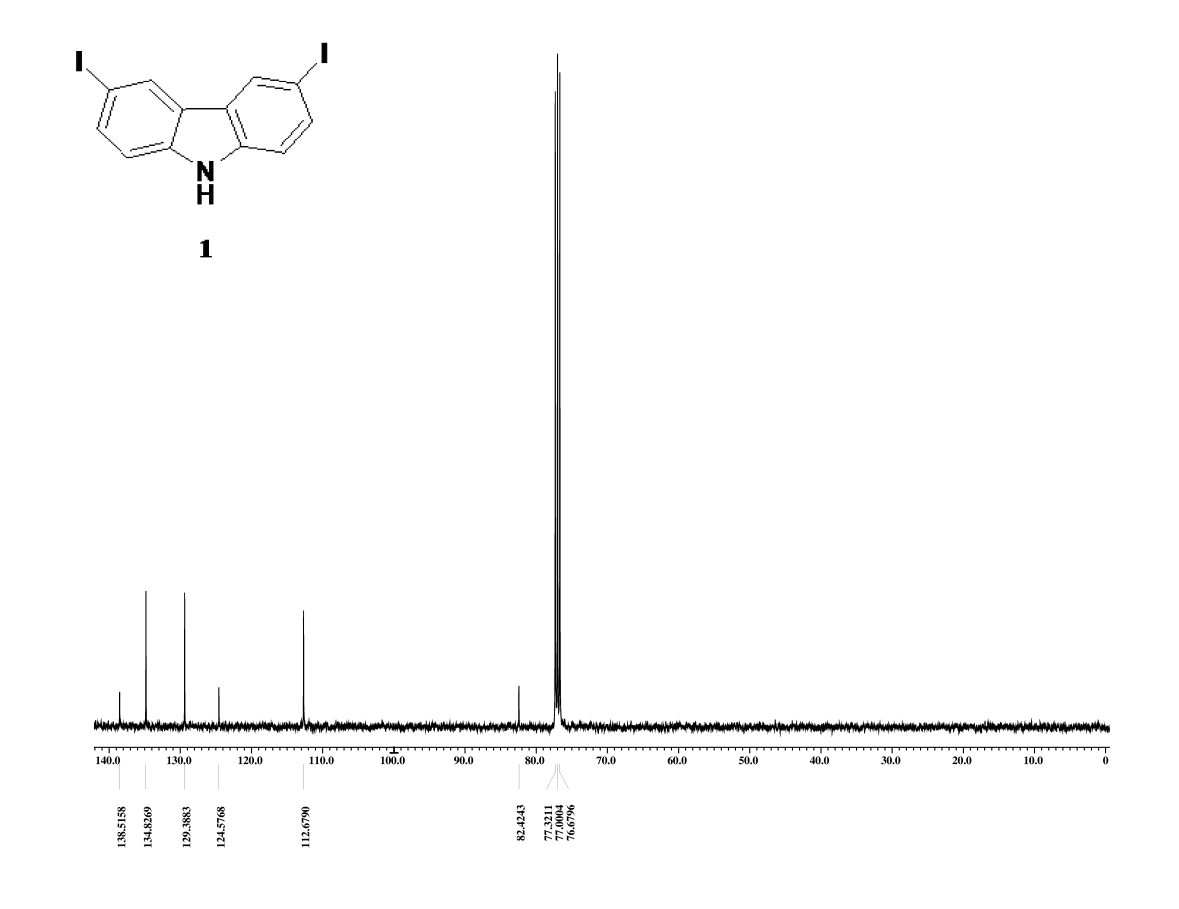
**

**^1^H and ^13^C NMR of 3:**


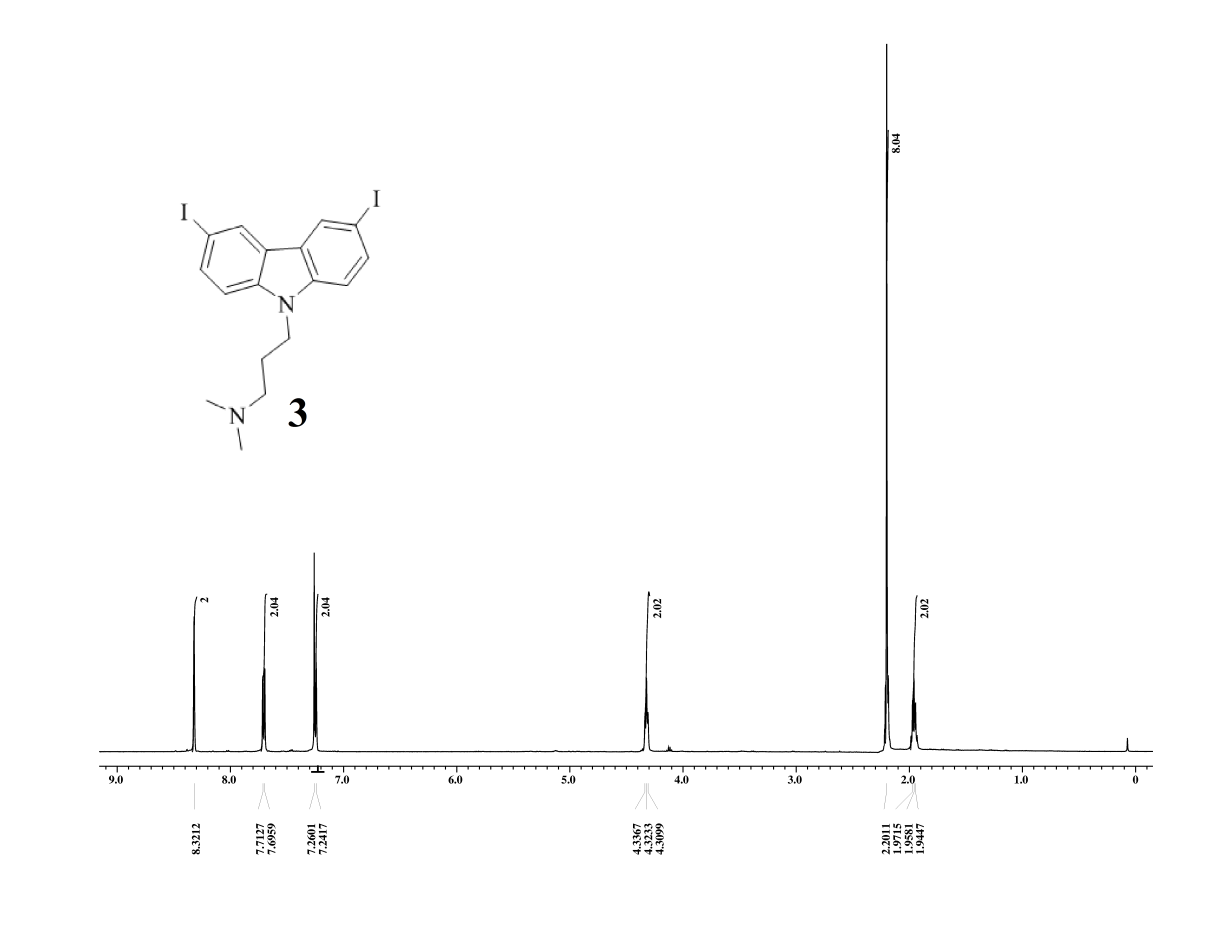


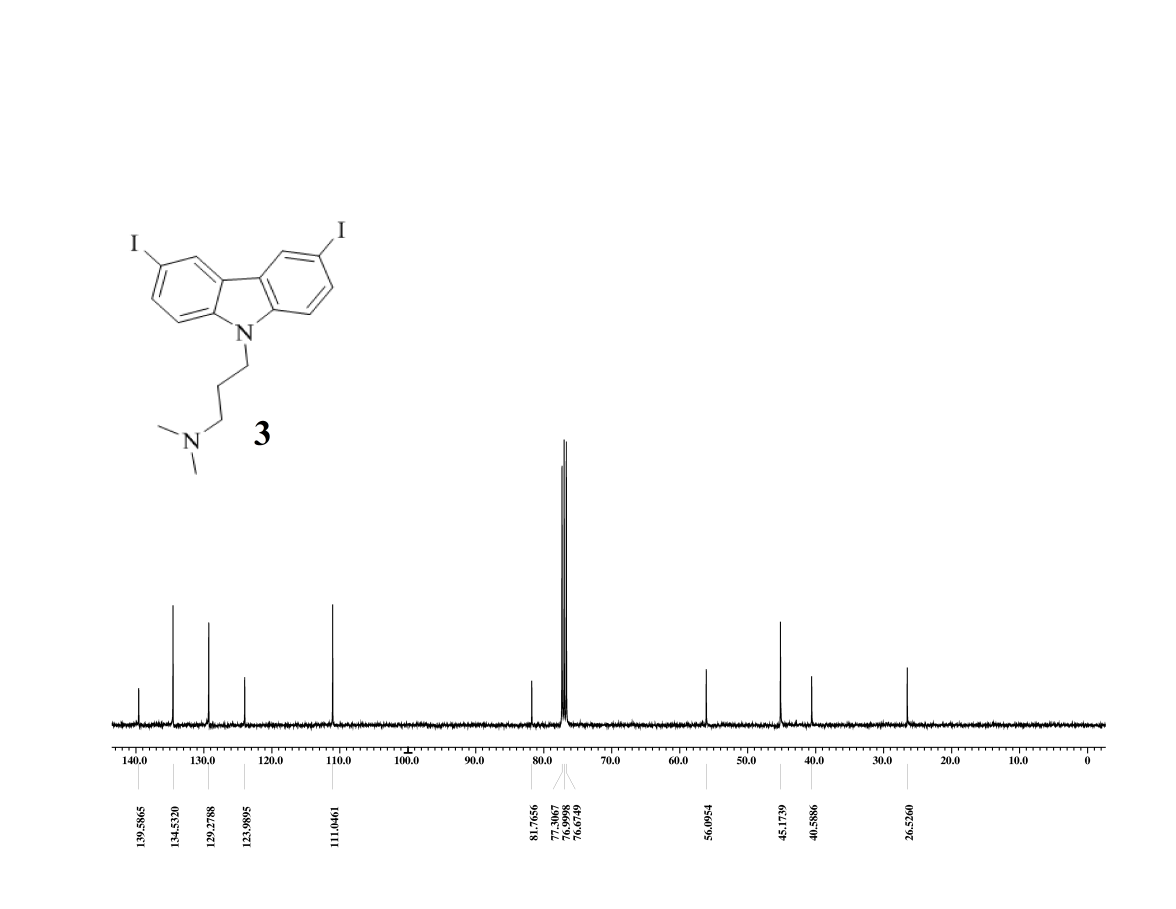


**^1^H and ^13^C NMR of 5:**


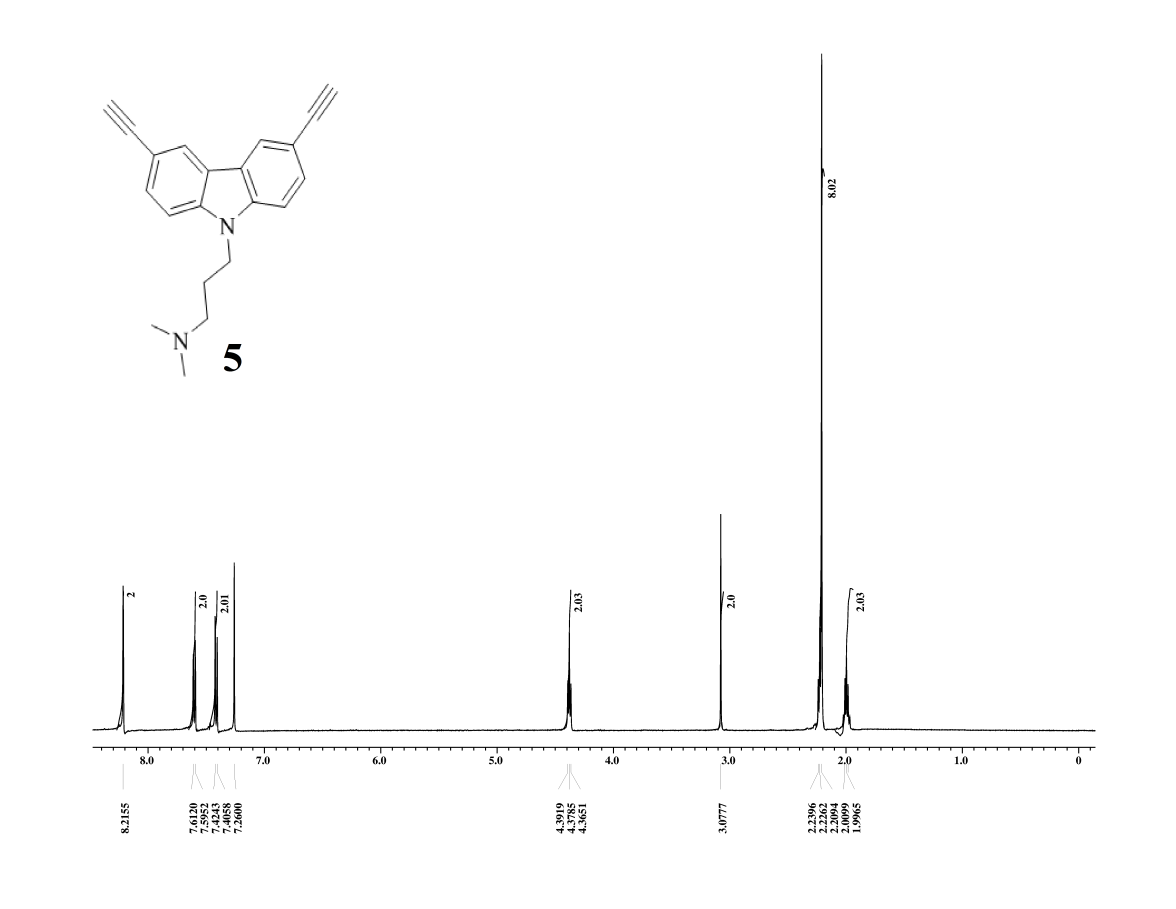

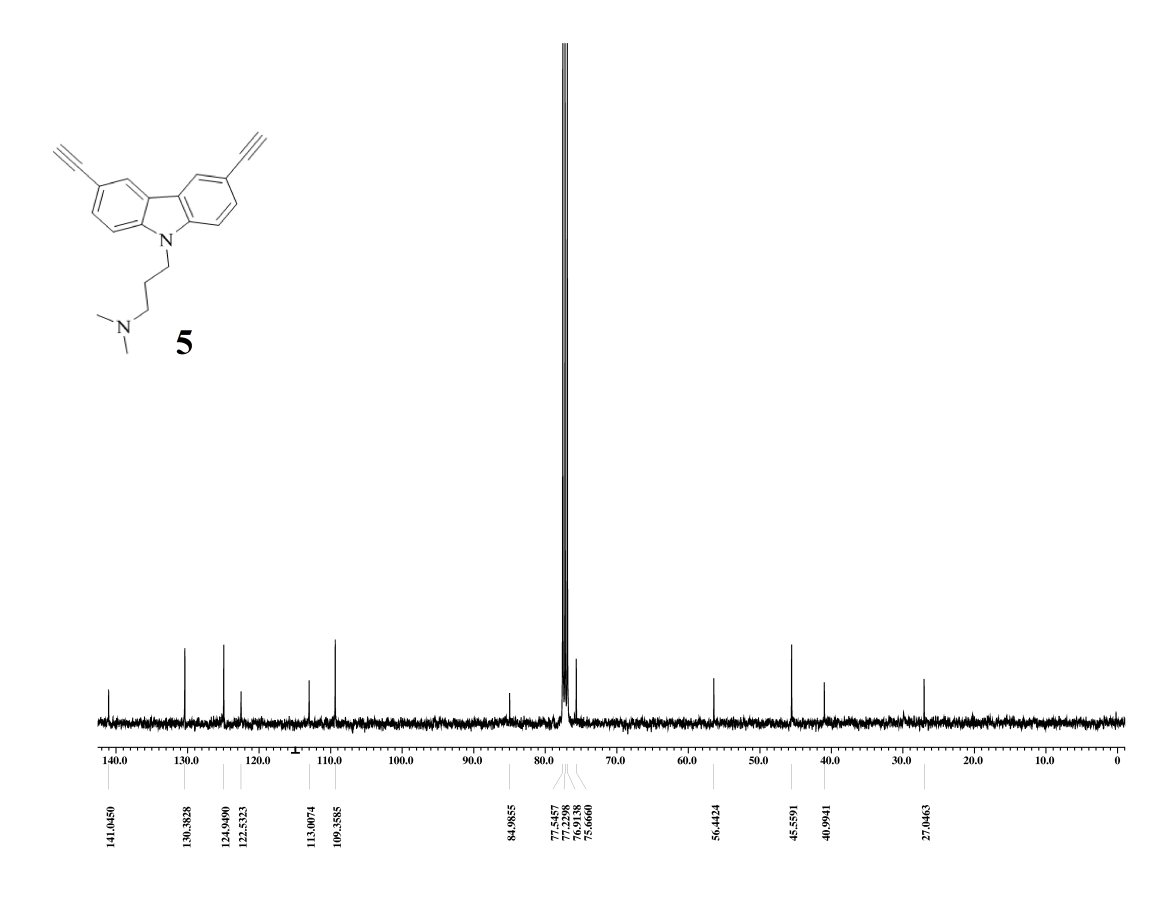


**^1^H and ^13^C NMR of 6f:**

**
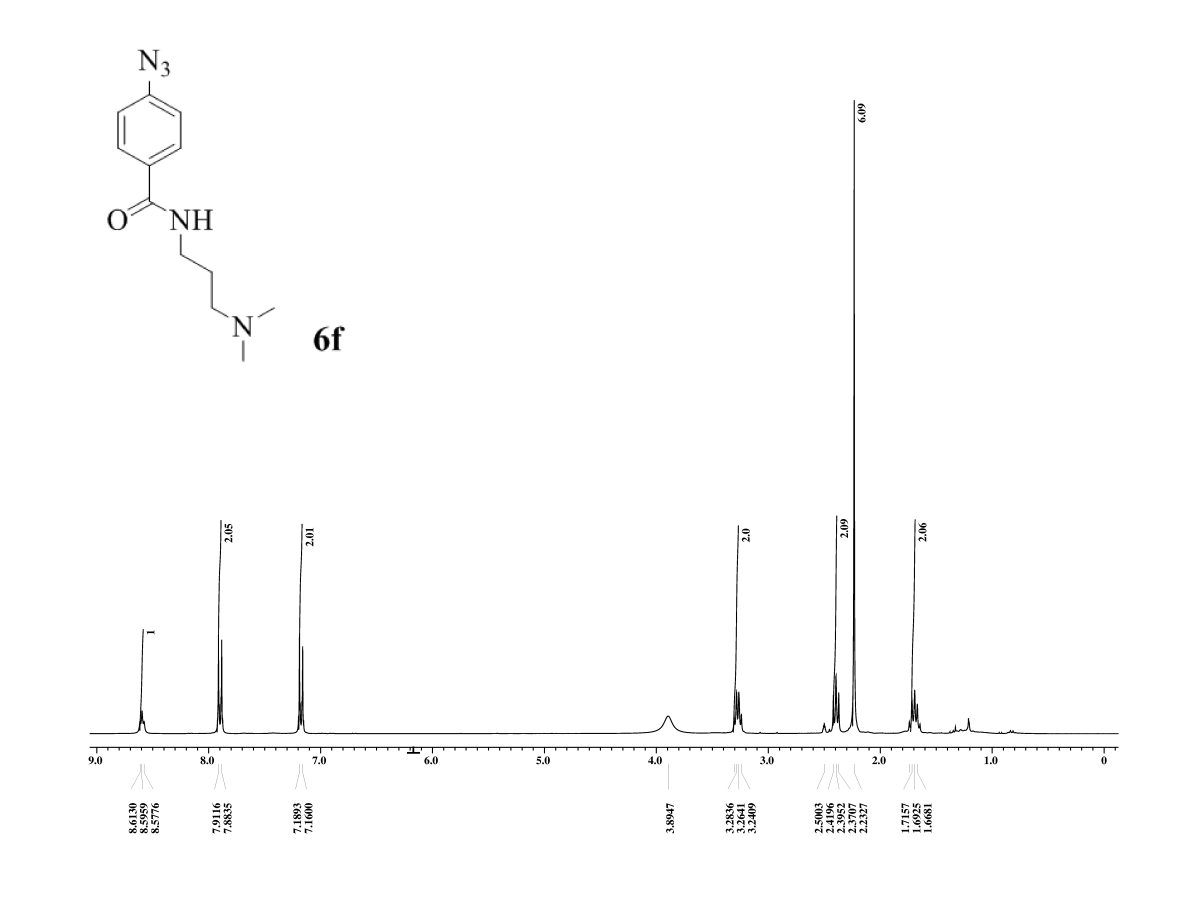
**

**
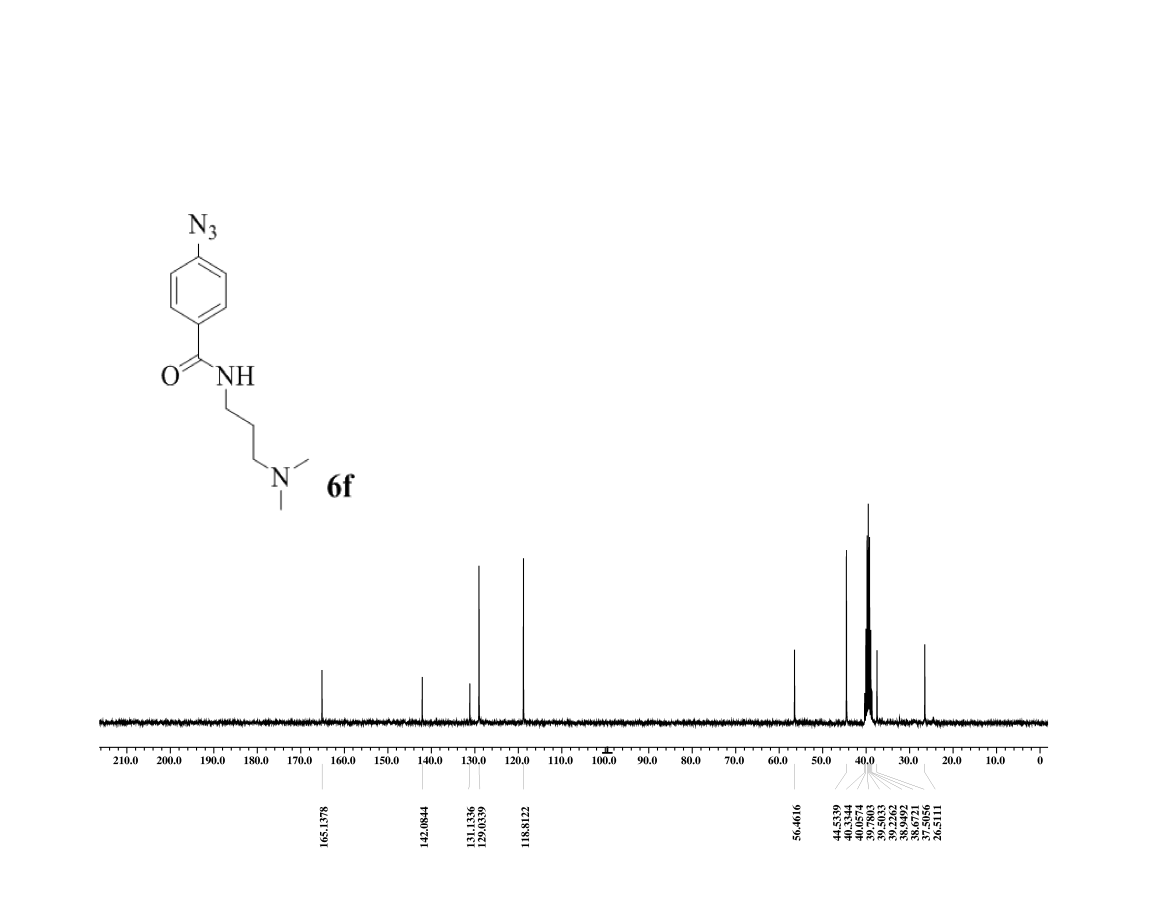
**

**^1^H and ^13^C NMR of BTC-a:**

**
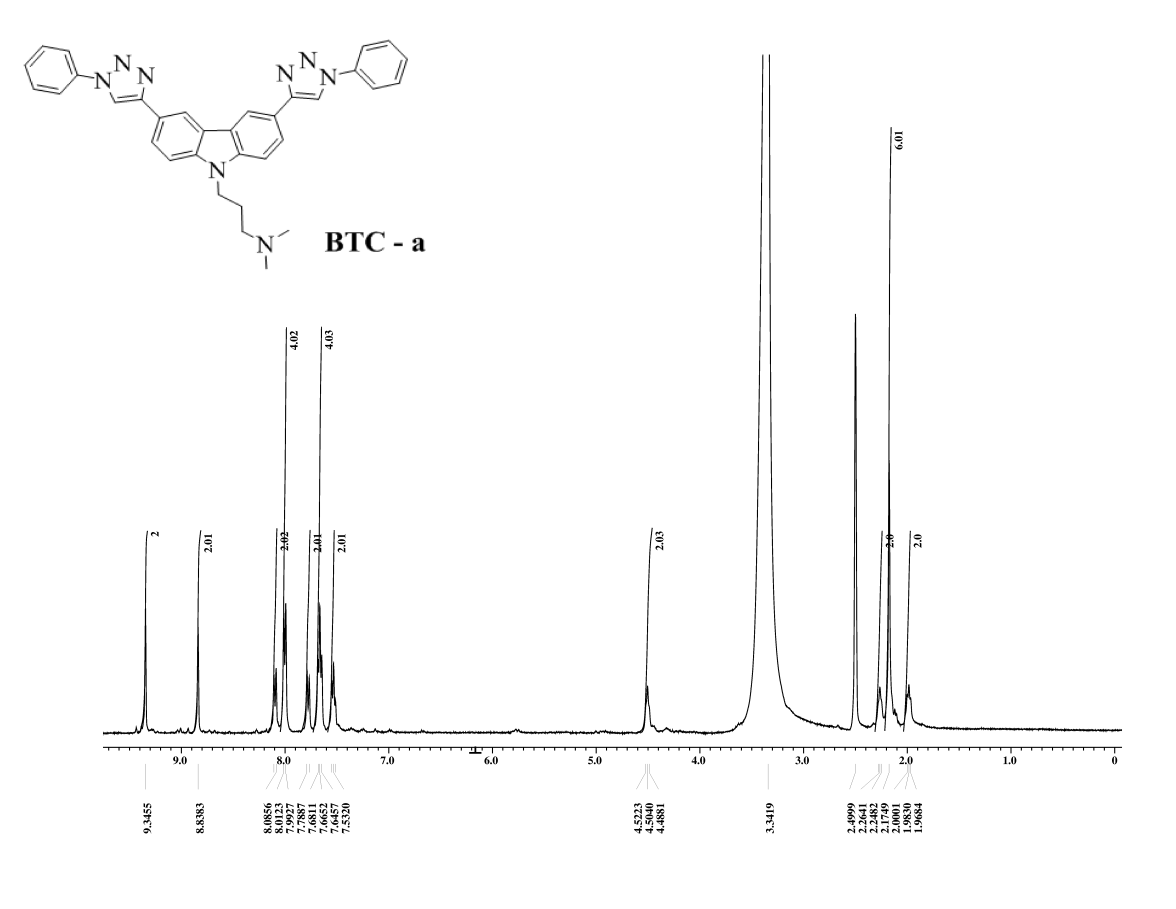
**
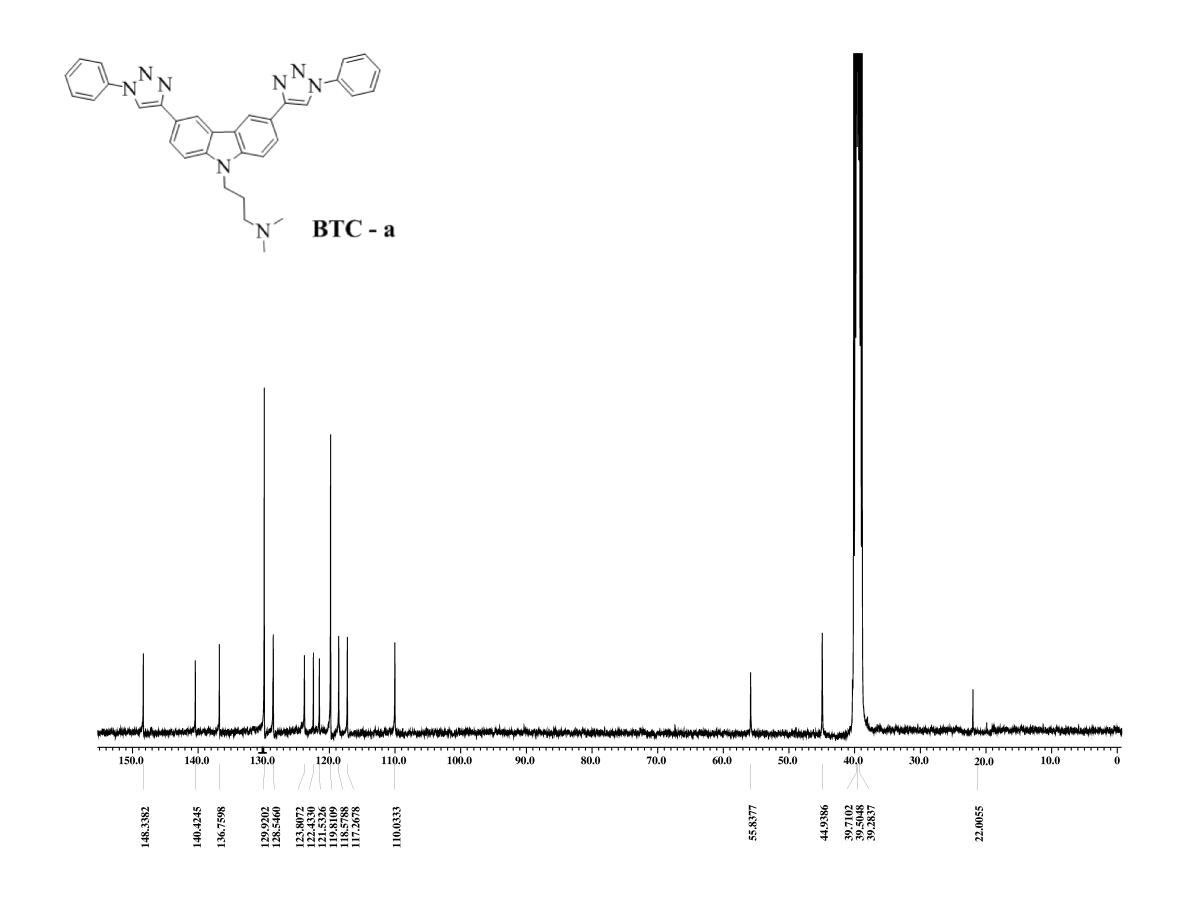


**^1^H and ^13^C NMR of BTC-b:**

**
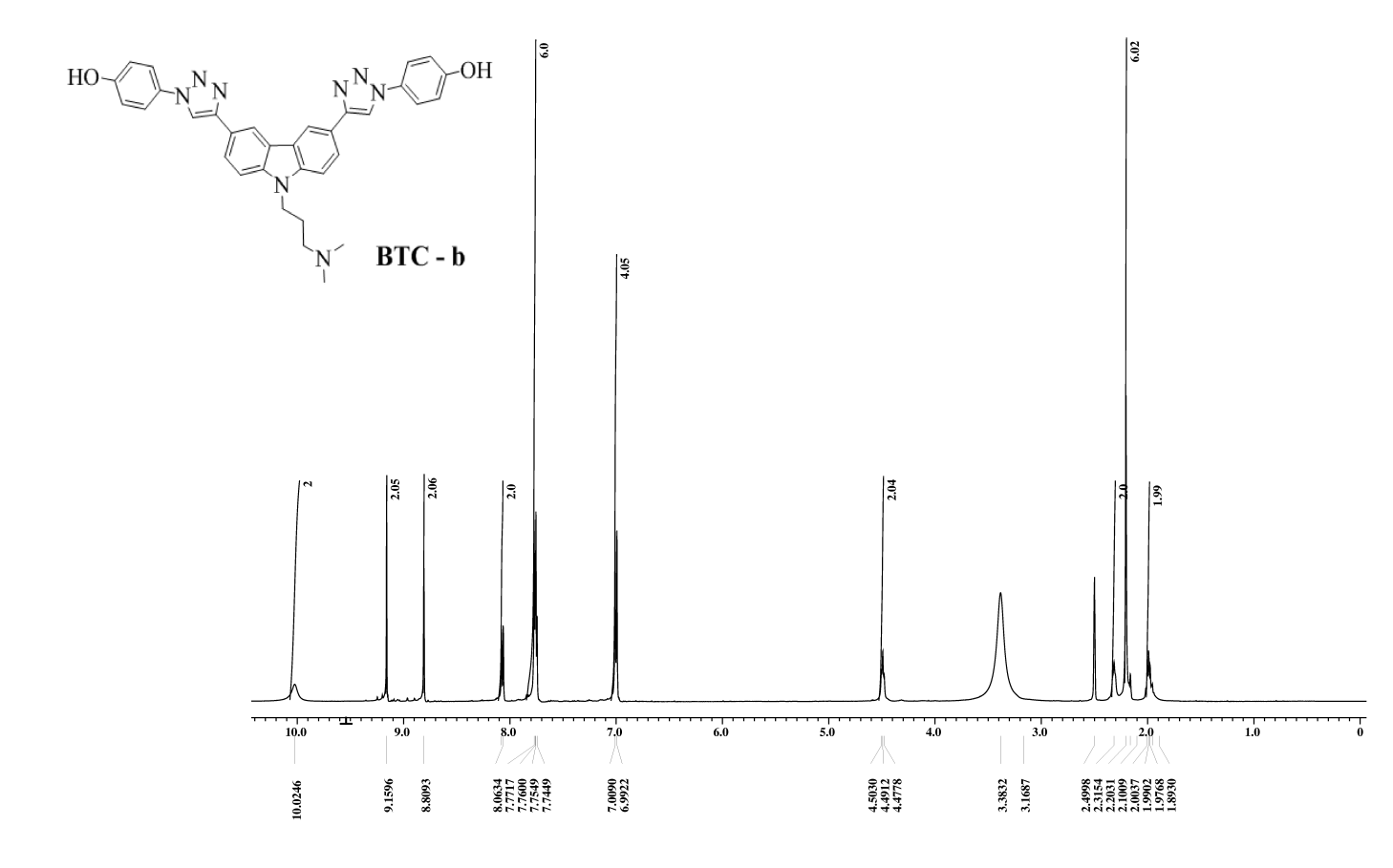
**


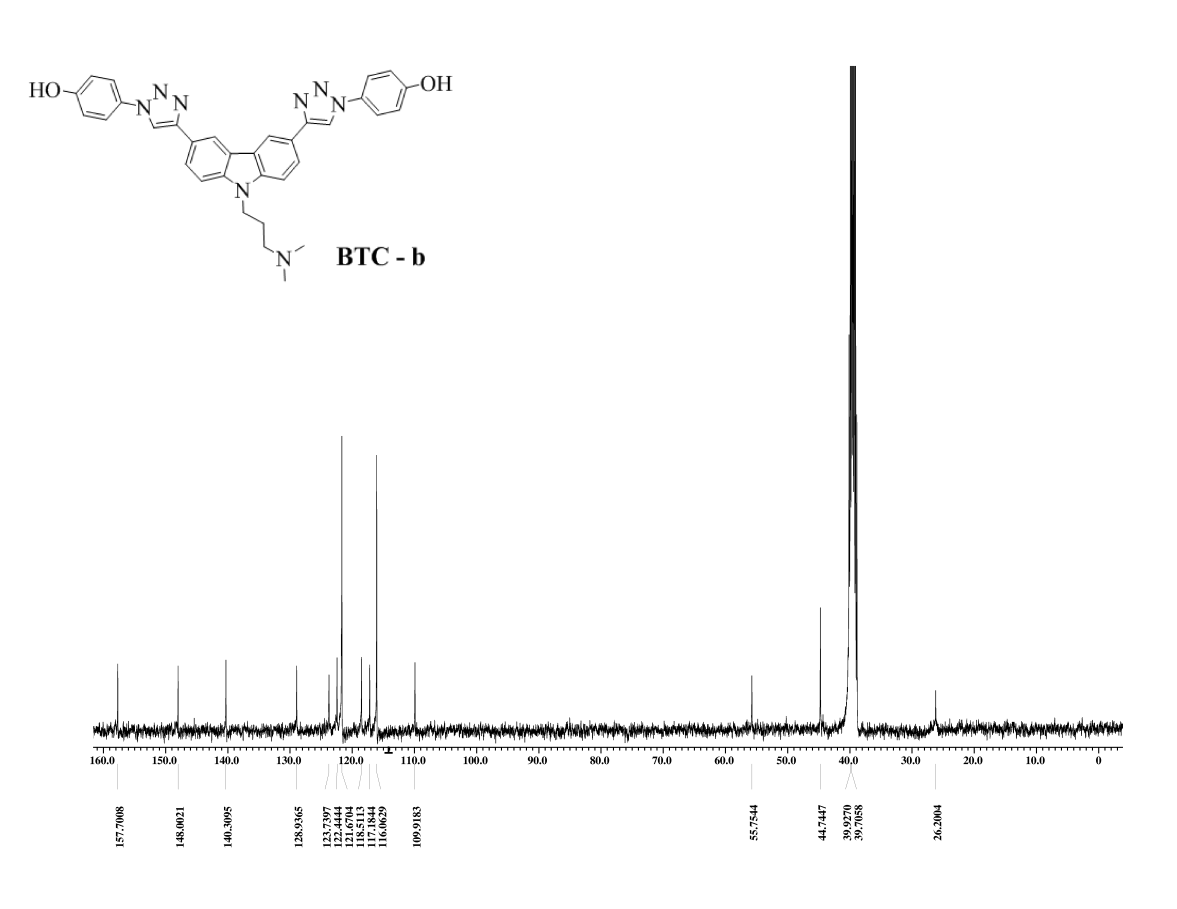


**^1^H and ^13^C NMR of BTC-c:**

**
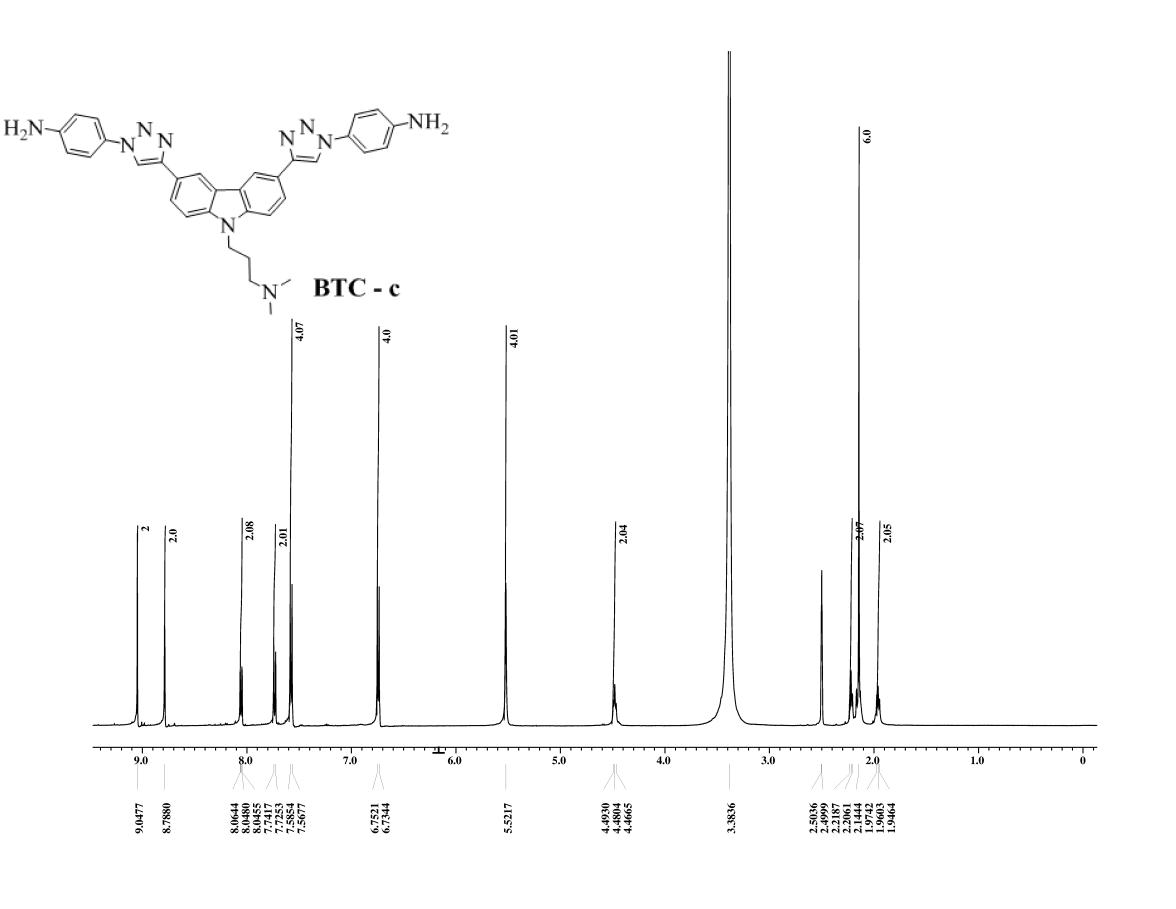
**


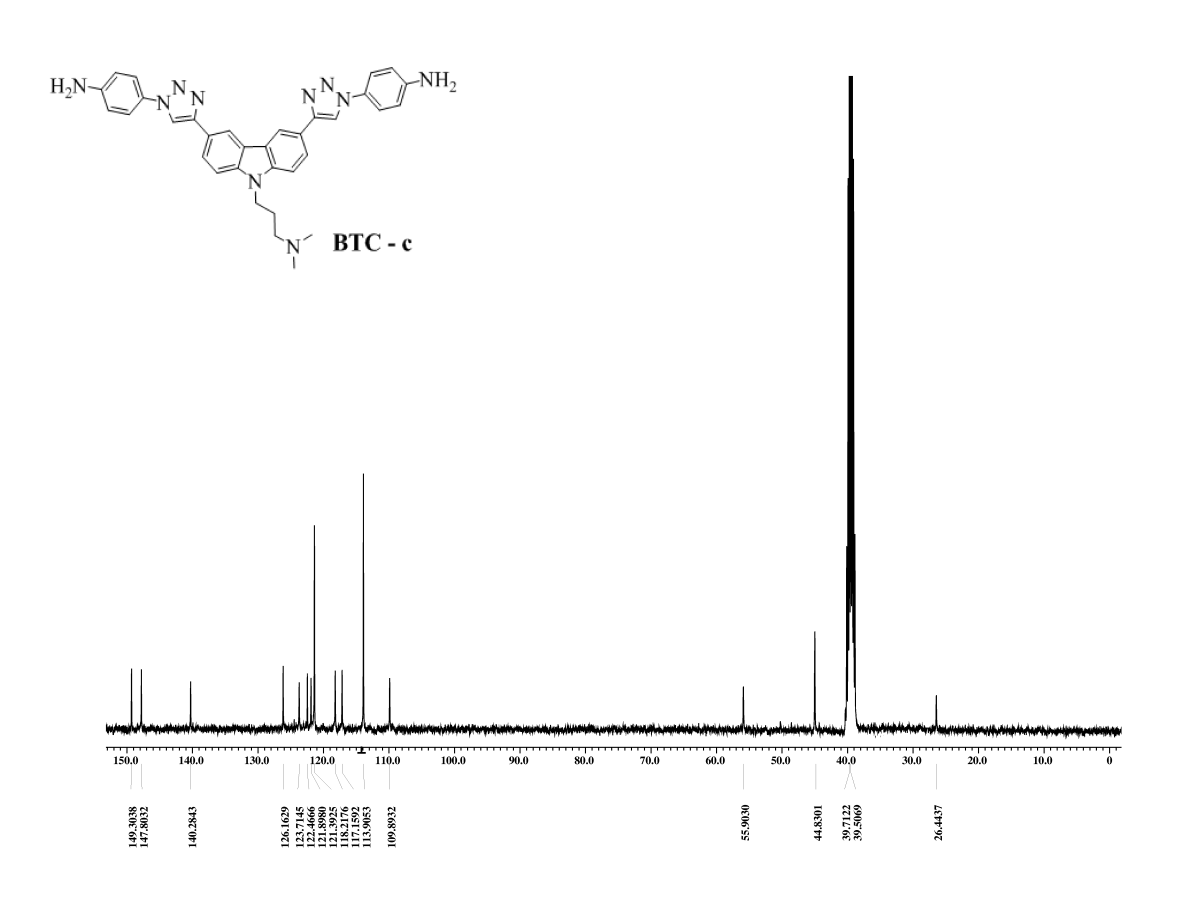


**^1^H and ^13^C NMR of BTC-d:**

**
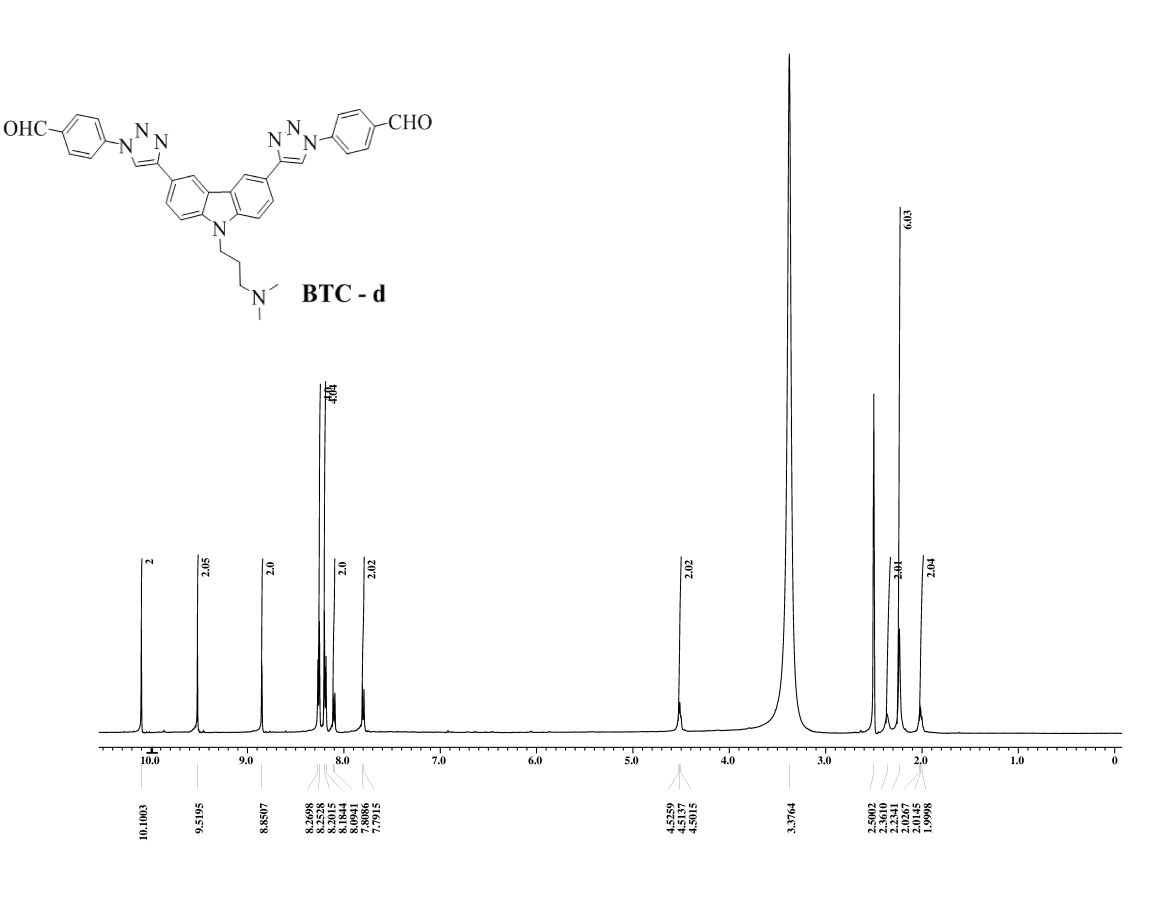
**

**
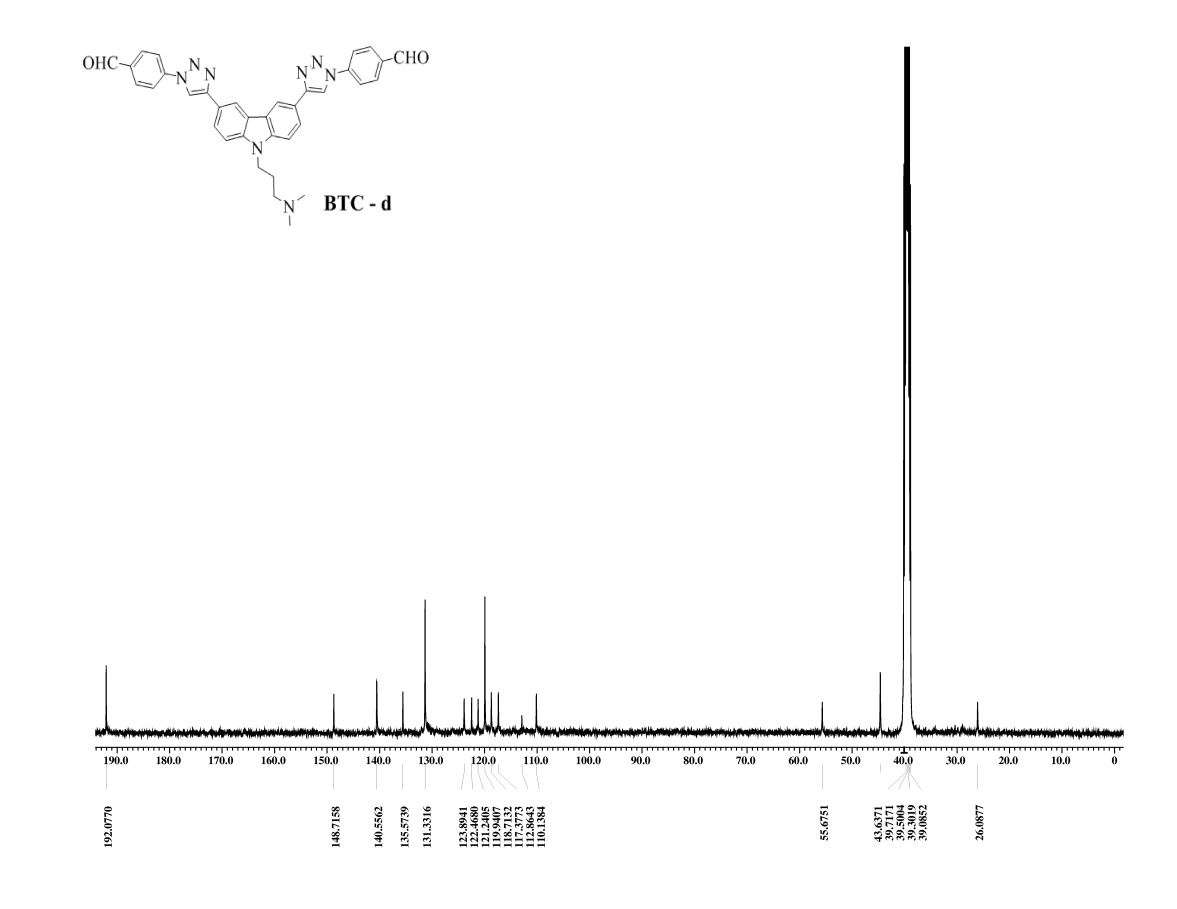
**

**^1^H and ^13^C NMR of BTC-e:**

**
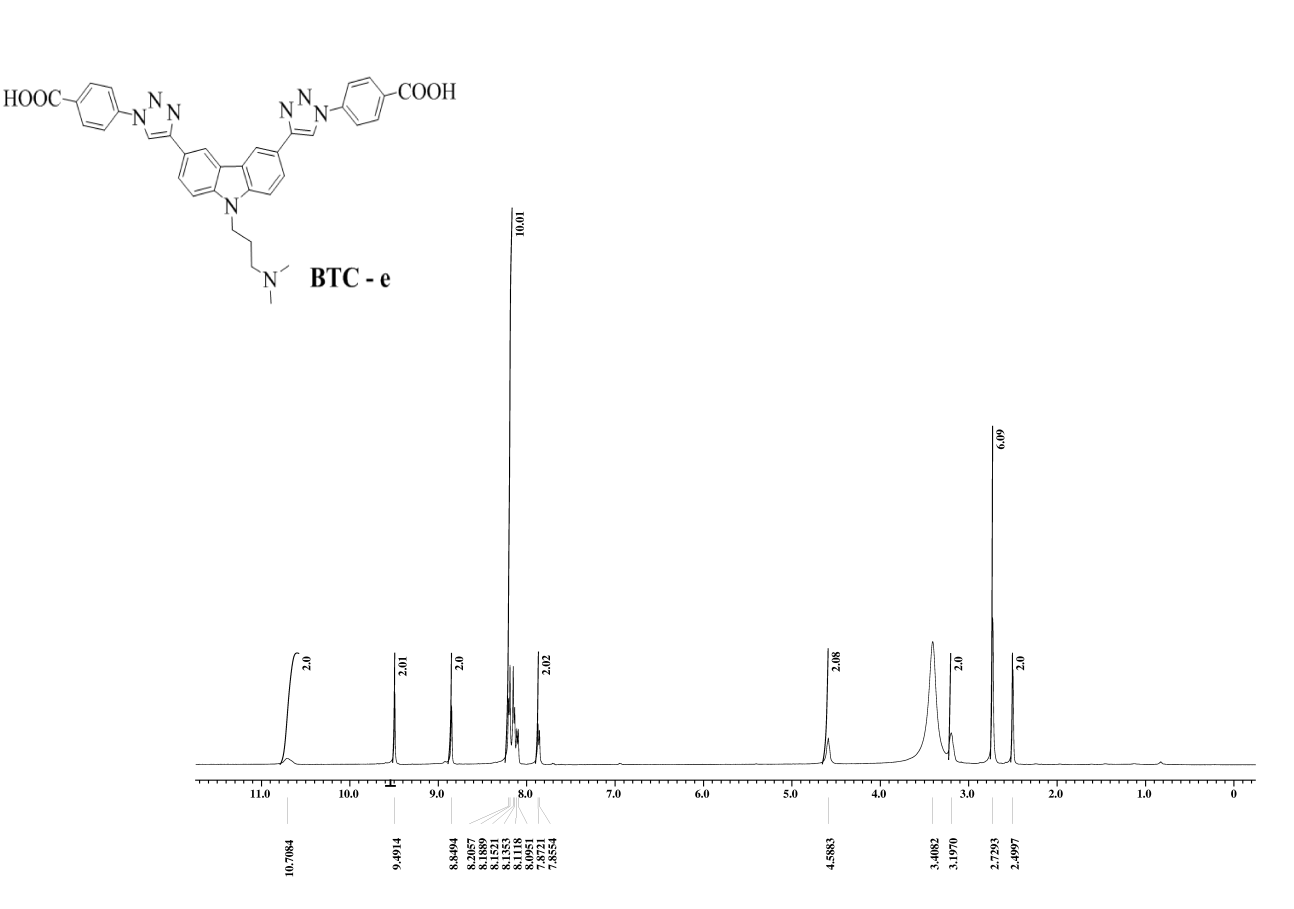
**

**
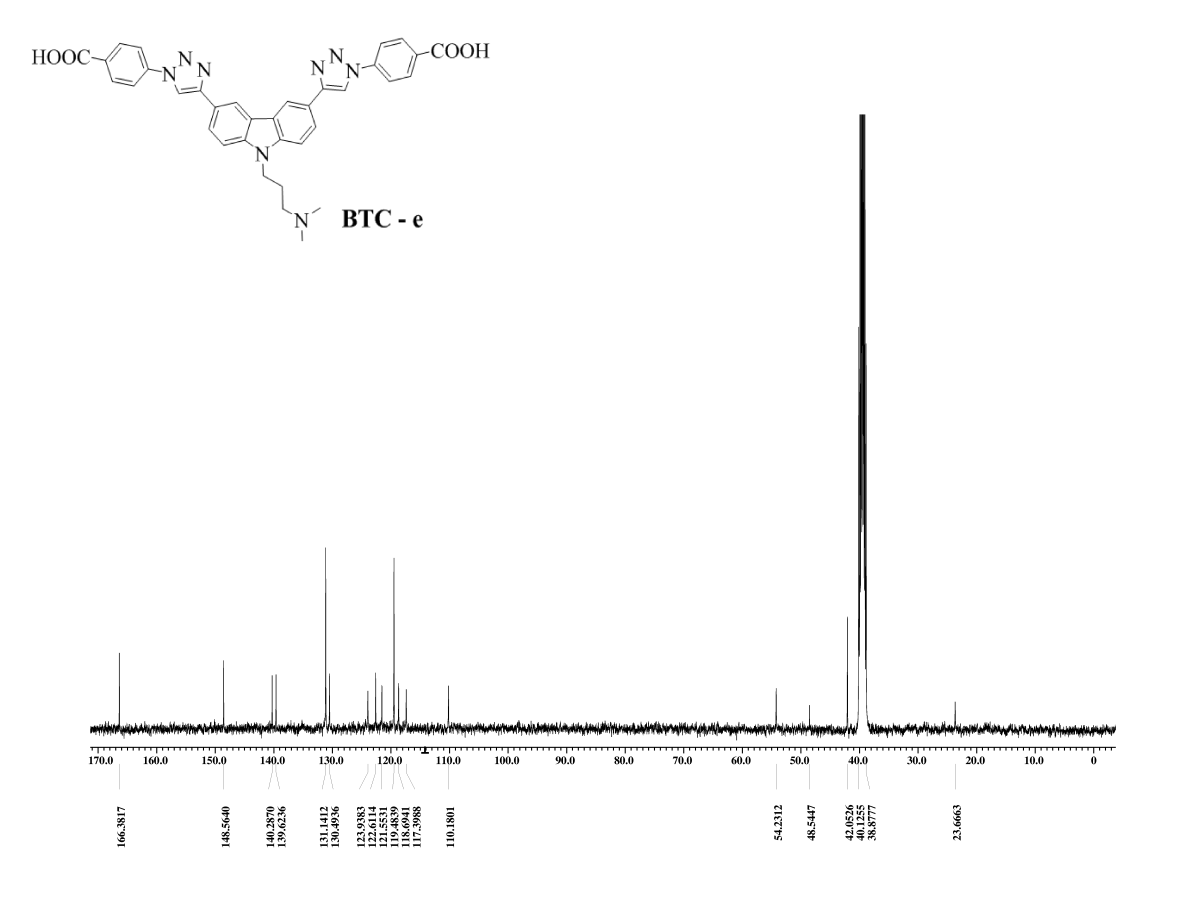
**

**^1^H and ^13^C NMR of BTC-f:**

**
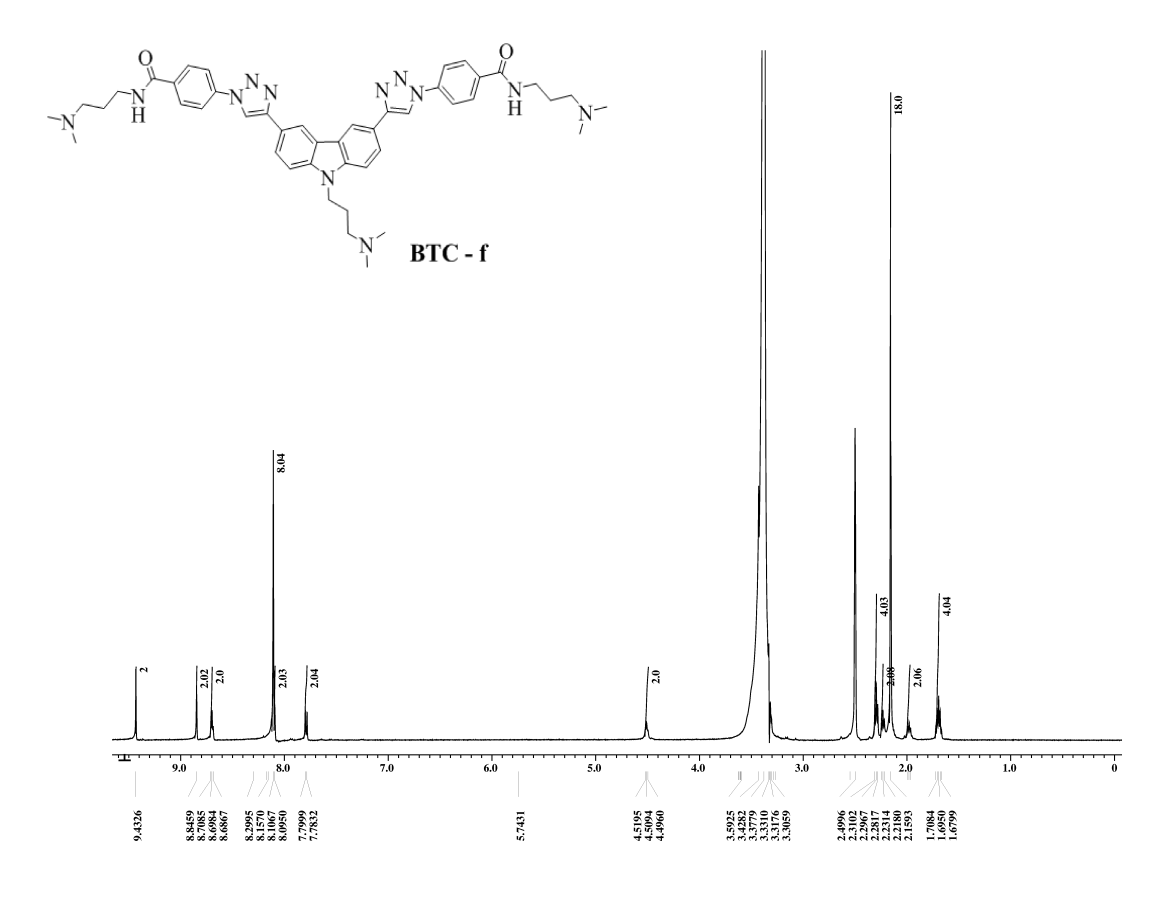
**

**
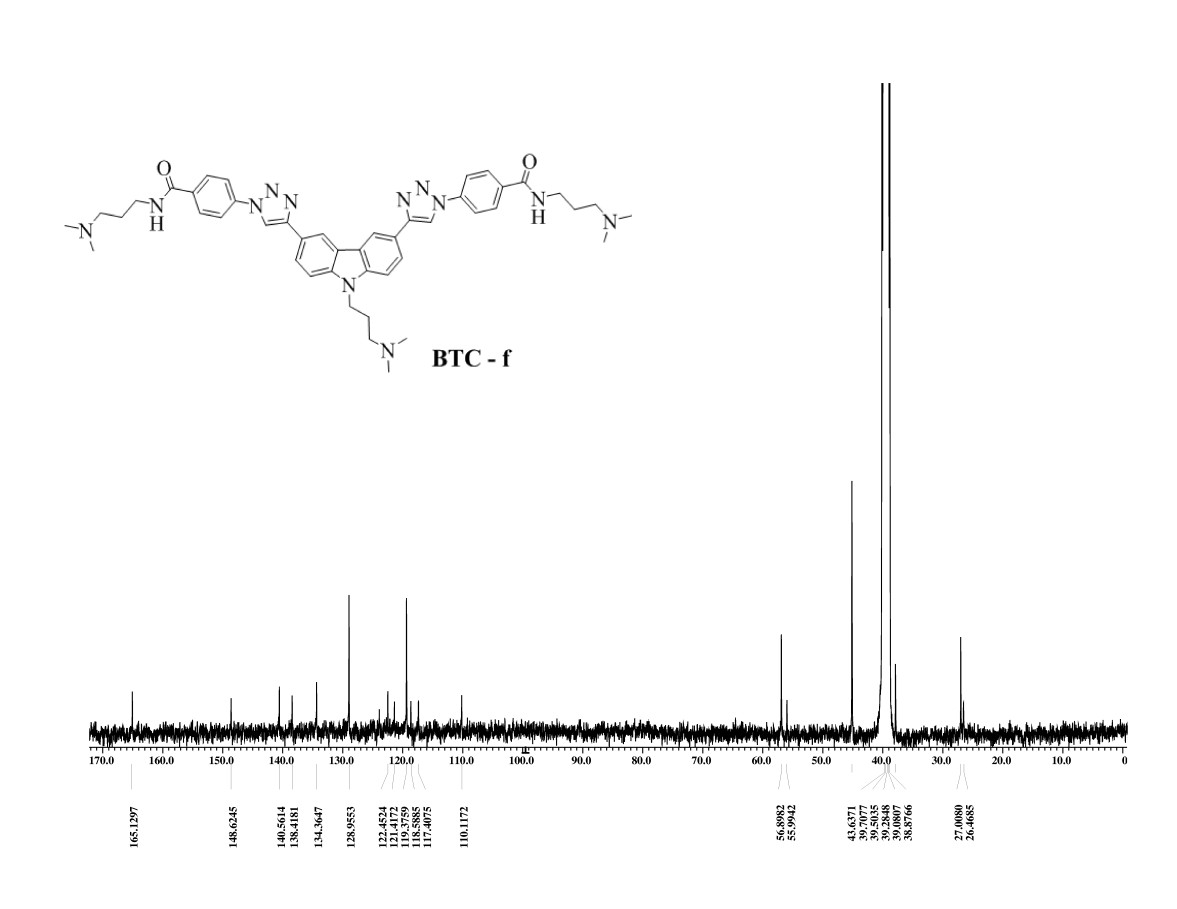
**

**^1^H and ^13^C NMR of BTC-g:**

**
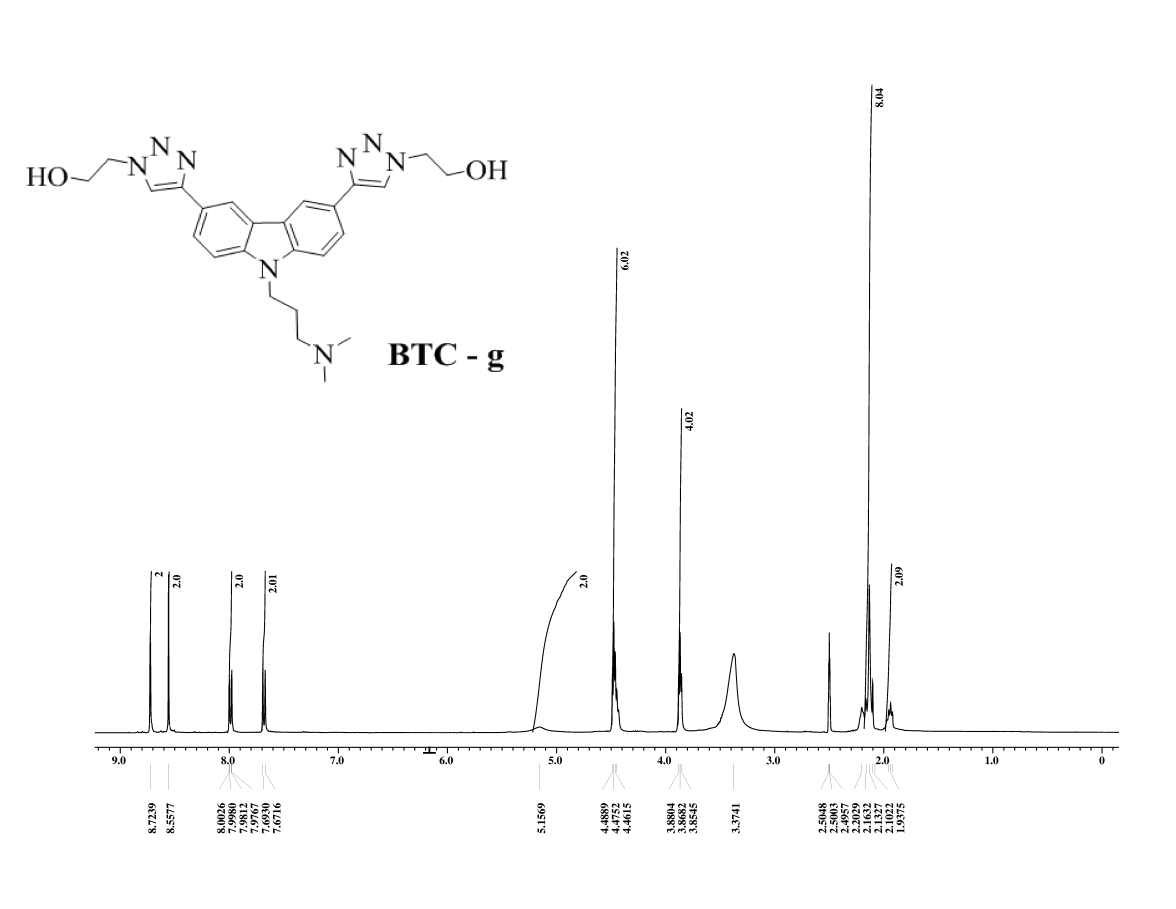
**

**
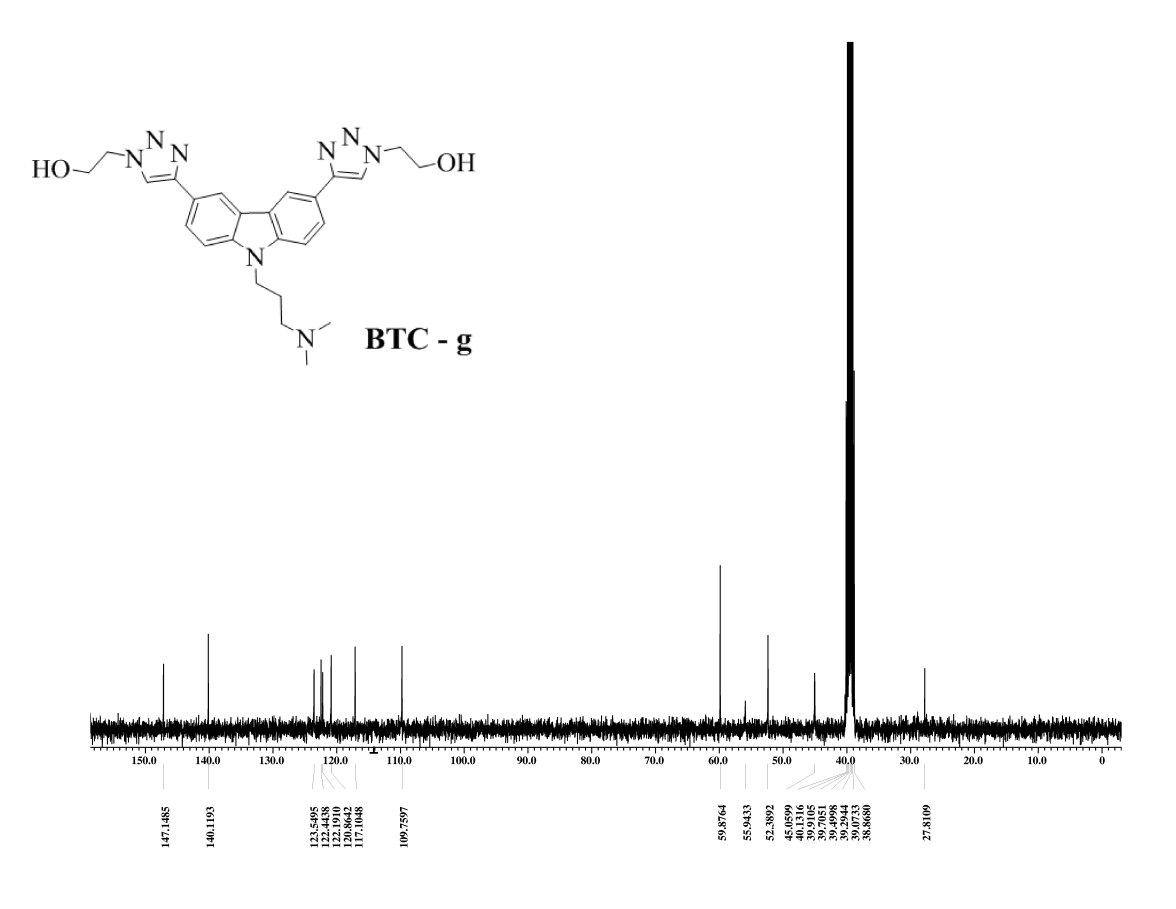
**

**10.0 References**

1. Berry, M. T., Castrejon, D., Hein, J. E. Oxidative esterification of aldehydes using mesoionic 1,2,3-triazolyl carbene organocatalysts*. Org. Lett*. **16,** 3676–3679 (2014).
2. Rena, L., Jiao, N. PdCl_2_ catalyzed efficient assembly of organic azides, CO, and alcohols under mild conditions: a direct approach to synthesize carbamates. *Chem. Commun.* **50,** 3706–3709 (2014).
3. Paladhi,S., Das, J., Mishra, P. K., Dash, J. Multifunctional “click” prolinamides: a new platform for asymmetric aldol reactions in the presence of water with catalyst recycling. *Adv. Synth. Catal*. **355,** 274–280 (2013).
4. Guldi, D. M. Triazole bridges as versatile linkers in electron donor acceptor conjugates*. J. Am. Chem. Soc*. **133,** 13036–13054 (2011).
5. [Pagoti](http://pubs.rsc.org/en/results?searchtext=Author%3ASreenivasarao Pagoti), S., [Surana](http://pubs.rsc.org/en/results?searchtext=Author%3ASubham Surana), S., [Chauhan](http://pubs.rsc.org/en/results?searchtext=Author%3AAjay Chauhan), A., [Parasar](http://pubs.rsc.org/en/results?searchtext=Author%3ABibudha Parasar), B., [Dash](http://pubs.rsc.org/en/results?searchtext=Author%3AJyotirmayee Dash), J. Reduction of organic azides to amines using reusable Fe_3_O_4_ nanoparticlesin aqueous medium. ***Catal. Sci. Technol.*** **3**, 584–588 (2013).
6. Wu, J.C., Wang, D.X., Huang, Z.T., Wang, M. X. Synthesis of diverse N,O-bridged calix[1]arene[4]pyridine-C60 dyads and triads and formation of intramolecular self-inclusion complexes*. J. Org. Chem*. **75**, 8604–8614 (2010).
